# Development of Nano-Antifungal Therapy for Systemic and Endemic Mycoses

**DOI:** 10.3390/jof7020158

**Published:** 2021-02-23

**Authors:** Jorge H. Martínez-Montelongo, Iliana E. Medina-Ramírez, Yolanda Romo-Lozano, Antonio González-Gutiérrez, Jorge E. Macías-Díaz

**Affiliations:** 1 Department of Chemistry, Universidad Autónoma de Aguascalientes, Aguascalientes 20131, Mexico; jorge.martmont@gmail.com (J.H.M.-M.); jangogu05@gmail.com (A.G.-G.); 2 Department of Microbiology, Universidad Autónoma de Aguascalientes, Aguascalientes 20131, Mexico; yolanda_rloza@hotmail.com; 3 Department of Mathematics, School of Digital Technologies, Tallinn University, 10120 Tallinn, Estonia; jemacias@correo.uaa.mx; 4 Department of Mathematics and Physics, Universidad Autónoma de Aguascalientes, Aguascalientes 20131, Mexico

**Keywords:** copper (I) iodide, composites, antifungal, chitosan, atomic force microscopy

## Abstract

Fungal mycoses have become an important health and environmental concern due to the numerous deleterious side effects on the well-being of plants and humans. Antifungal therapy is limited, expensive, and unspecific (causes toxic effects), thus, more efficient alternatives need to be developed. In this work, Copper (I) Iodide (CuI) nanomaterials (NMs) were synthesized and fully characterized, aiming to develop efficient antifungal agents. The bioactivity of CuI NMs was evaluated using *Sporothrix schenckii* and *Candida albicans* as model organisms. CuI NMs were prepared as powders and as colloidal suspensions by a two-step reaction: first, the CuI${}_{2}$ controlled precipitation, followed by hydrazine reduction. Biopolymers (Arabic gum and chitosan) were used as surfactants to control the size of the CuI materials and to enhance its antifungal activity. The materials (powders and colloids) were characterized by SEM-EDX and AFM. The materials exhibit a hierarchical 3D shell morphology composed of ordered nanostructures. Excellent antifungal activity is shown by the NMs against pathogenic fungal strains, due to the simultaneous and multiple mechanisms of the composites to combat fungi. The minimum inhibitory concentration (MIC) and minimum fungicidal concentration (MFC) of CuI-AG and CuI-Chitosan are below 50 μg/mL (with 5 h of exposition). Optical and Atomic Force Microscopy (AFM) analyses demonstrate the capability of the materials to disrupt biofilm formation. AFM also demonstrates the ability of the materials to adhere and penetrate fungal cells, followed by their lysis and death. Following the concept of safe by design, the biocompatibility of the materials was tested. The hemolytic activity of the materials was evaluated using red blood cells. Our results indicate that the materials show an excellent antifungal activity at lower doses of hemolytic disruption.

## 1. Introduction

Fungal infections and fungal contamination have become an important worldwide public health problem with a considerable impact on human morbidity and mortality. Immunosuppression, cancer treatment, and immunological dysfunctions result in disseminated or systemic fungal diseases, that might evolve into a life-threatening condition. In recent years, mycoses have increased their incidence, thus, the development of novel targeted therapeutics is an important challenge to treat them. Changes in etiology, including the emergence of new pathogens, resistance to antifungals, and opportunistic and immunosuppressive factors that many patients experience, add to the difficulties in diagnosing and treating fungal infections, a fact that increases costs in the health sector [1].

*Sporothrix schenckii* is a widely distributed dimorphic fungus that is mainly recovered from environmental samples (plant or soil). It can cause sporotrichosis, which is a subcutaneous mycosis, with a subacute or chronic course in humans and other animals, and like the fungus, it has a wide geographical distribution. The infection usually occurs because of the traumatic inoculation of fungal conidia or fragments of hyphae, corresponding to its mycelial morphotype, into the skin or subcutaneous tissue. The clinical outcome of the infection depends on the immune response of the host, resulting in severe disseminated disease with visceral and osteoarticular involvement in immunocompromised individuals, particularly people with AIDS [2]. In infected tissue, the fungus differentiates into the pathogenic yeast form and may spread to other tissue. In addition, it can form resistance structures such as biofilms [3]. The gold standard for the treatment of the subcutaneous disease is itraconazole, although, in Mexico and some other underdeveloped countries, potassium iodide is still used, due to its low cost and safety.

*Candida albicans* is a pathogenic and opportunistic fungus responsible for numerous diseases. It is one of the most common causes of hospital-acquired systemic infections due to its adhesive and invasive properties, and its capability to form biofilms. The capacity of *C. albicans* to form biofilms increases their resistance to antifungal therapy and the ability of the yeast cells within the biofilm consortium to withstand host immune response [4]. Miconazole and fluconazole are commonly used for candidiasis treatment; however, side deleterious effects, fungal resistance, and relapse cases have been observed with the use of these anti-fungal agents.

As previously mentioned, antifungal therapy for systemic mycosis is limited, most of the time expensive and causes important toxic effects. Nanotechnology has become an interesting strategy to improve the efficacy and specificity of traditional antifungal drugs, since it allows lower toxicity, better bio-distribution, drug targeting, potent activity, and broad antifungal spectrum. Nanotechnology has positively impacted the advances in the development of novel strategies for the cure of infectious diseases. For instance, it has been demonstrated that trypanocidal therapy based on nano-systems, renders higher accessibility, improved selectivity, and specific delivery of the active principle to intracellular targets [5]. Different nanomaterials have been evaluated for the development of antimicrobial agents: Metallic nanoparticles (Ag, Cu, Au, Al), metal oxides (ZnO, TiO${}_{2}$, Fe${}_{3}$O${}_{4}$, CoFe${}_{2}$O${}_{4}$), fullerenes, carbon nanotubes, antimicrobial peptides and chitosan [6]. Lately, the use of CuI NPs was shown to inhibit the growth of Gram (+) and Gram (-) bacteria [7].

Biopolymers are valuable and flexible materials for biomedical and pharmaceutical applications. Their use as an excipient has increased since stable colloidal suspensions of different nanostructured materials can be formulated. In addition to the colloidal stability, the biopolymer might enhance the therapeutic efficacy of a compound. For example, chitosan composites are of interest due to the bioactivity of the polymer [8]. Chitosan has been investigated for its antimicrobial activity. This is shown by its multiple mechanisms: damage to peptidoglycan and/or cell membrane, damage to DNA or inhibition on the synthesis of mRNA, decreases the metalloproteinase activity of the microorganisms by metal chelation [9]. Formulations that involve the use of chitosan and nanoparticles exhibit increased surface area. Also, at physiological pH values, an increased density of positive charge on the surface of the composite favors the interaction to negatively charged biomolecules on the cell walls of microorganisms. This increases its antimicrobial activity. In this study, Copper (I) Iodide (CuI)-Arabic Gum (CuI@AG) and CuI-Chitosan (CuI@Ch) composites were prepared and evaluated for the inhibition of biofilm-forming pathogenic fungi (*S. schenckii*, *C. albicans*).

CuI is an interesting material that has found important industrial and biomedical applications [7,10,11]. Previous studies from our research group have demonstrated the antimicrobial activity of different metal oxide nanostructured materials [12,13,14,15,16,17,18]. We have also reported that the toxicity of metallic nanostructures is partly due to the lixiviation of ions to the medium, observing a direct correlation of toxicity and ion concentration. We explore the use of hybrid materials, to reduce toxicity and increase bio-activity (in this particular case, anti-fungal activity). In this work, we report on the synthesis, characterization, and antifungal activity of CuI composites. The composite under study contains three elements (Cu, I, chitosan) that show antifungal activity, which facilitates their administration at low doses. Moreover, the components of the composite exhibit activity under different mechanisms limiting the capacity of the pathogen to overcome their bioactivity.

## 2. Materials and Methods

### 2.1. Materials and Reagents

Copper sulfate pentahydrate (Karal, León, Mexico), potassium iodide (J.T. Baker, Estado de Mexico, Mexico), hydrazine hydrate (Sigma-Aldrich, St. Louis, MO, USA, 60%) chitosan (Sigma-Aldrich, Reykjavik, Iceland, medium molecular weight, deacetylation ≥75%, viscosity 200–800 cP, 1 wt% in 1% acetic acid, 25 ${}^{\circ }$C), Arabic gum (Golden Bell, Zapopan, Mexico), Agar dextrose sabouraud (BD Bioxon, Estado de Mexico, Mexico), potato dextrose agar (BD Bioxon, Estado de Mexico, Mexico), were all analytical grade and used as received.

### 2.2. Synthesis of CuI Nanostructured Materials

CuI nanostructured materials were prepared by a modification in the method reported by Pramanik et al. [7]. In brief, a 6.0 mM potassium iodide solution was added to 6.0 mM of copper sulfate solution containing 5% (*w*/*v*) Arabic gum as a stabilizing agent. After that, a 30 μL concentration of hydrazine were dropwise added to the above mixture and the reaction was continued for 1 h at 60 ${}^{\circ }$C. The nanostructured materials were collected by centrifugation at 4000 rpm for 10 min. These particles were washed several times with deionized water followed by centrifugation. The CuI NPs were recovered and dried at 60 ${}^{\circ }$C in hot air oven for 6 h. These particles were fairly stable at room temperature for 1–2 weeks. They were stable in the sense that Cu did not oxidize and the composite did not aggregate (precipitate) during those 1–2 weeks. For the CuI@Ch composites, a 1.5% (*w*/*v*) solution of Medium Molecular Weight (MMW) chitosan was prepared in a 1% acetic acid solution. CuI nanostructured materials were prepared as powders and colloidal suspensions.

### 2.3. Characterization of CuI Nanostructured Materials

CuI nanostructured materials were characterized by scanning electron microscopy (SEM-EDX) (Carl Zeiss, Sigma-HDVP and EDX detector QUANTAX Brucker) and atomic force microscopy. Thin films of CuI@AG and CuI@Ch were deposited on a glass substrate using doctor blade technique. The surface roughness and morphology at micro- and nanometer scale were measured with a ScanAsyst atomic force microscope (Bruker, Dimension Edge with Scan Assist). The samples were analyzed in tapping mode in air with OTESPA-R3 tips (silicon; f${}_{0}$: 300 kHz; k: 26 N/m). Data was acquired on square frames of 10 × 10, 5 × 5 and 2.5 × 2.5 m. Images were recorded using height, amplitude and phase channels. Amplitude mode was used to evaluate the topography of the films; height mode images were used to obtain quantitative measurements, and phase images to evaluate changes in composition. A resolution of 512 × 512 pixels was used. Measurements were made by triplicate on different zones of each sample.

### 2.4. Evaluation of the Antifungal Activity of CuI Nanostructured Materials

**Figure 1:**
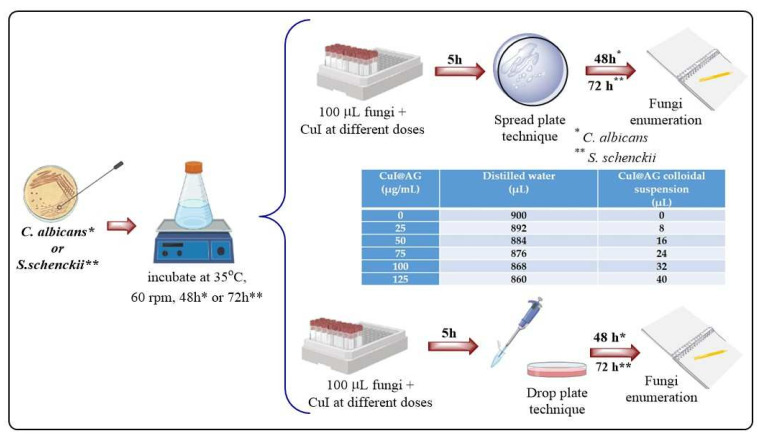
Schematic representation of the methodological approach for the evaluation of the antifungal activity of CuI@biopolymer against pathogenic fungi (*C. albicans*, *S. schenckii*).

**Fungal strains and culture conditions.** A general scheme for the antifungal evaluation of CuI@AG and/or CuI@Ch is illustrated in Figure 1. The antifungal activity of Cu@AG against *C. albicans* was used as a reference to demonstrate the enhanced activity of CuI@AG. The concentration of copper in the Cu NPs is the same as the concentration used for CuI NMs.

***Sporothrix schenckii*****.** The *S. schenckii* sensu stricto wild-type strain UAA-307 was obtained from a human lymphocutaneous sporotrichosis case. The fungus identification was previously performed by biochemical, morphological, and molecular biology techniques (polymerase chain reaction sequencing of the calmodulin gene, accession number KJ921740 in GenBank) [19]. Conidial suspensions were prepared as previously reported [20]. Briefly, fungal mycelium was incubated at 28 ${}^{\circ }$C for three days in sabouraud dextrose broth under orbital agitation at 150 rpm (Shaker Lumistell, IR0-60, Temperature Control). The fungal suspension was filtered on sterile filter paper (pore size 0.45 mm) and harvested by centrifugation at 750×*g*, for 20 min at 4 ${}^{\circ }$C. The fungal cells were washed then with sterilized phosphate buffer solution (PBS) and counted in a Neubauer chamber before all experiments. The bioavailability of the fungi was determined by trypan blue staining. In all the experiments, a stock fungal suspension of $1\times 10^{7}$ conidia/mL in distilled water was used.

***Candida albicans*****.***C. albicans* strain used in this study was a clinical isolate, and isolates were cultured in Sabouraud Dextrose Agar (SDA), then incubated at 37 ${}^{\circ }$C for 48 h. Fungal biomass was collected afterwards by a sterile loop from the surface of SDA medium and resuspended in 1 mL of PBS. An inoculum of *C. albicans* was placed in 3 mL of Potato Dextrose broth and left to grow in a shaking water bath (60 rpm) at 35 ${}^{\circ }$C for 48 h (resulting in $1\times 10^{7}$ CFU/mL according to the growth phase). The fungal suspension was centrifuged next at 3500 rpm for 5 min, decanted, and suspended in distilled water (stock fungal suspension).

***Fusarium oxysporum*****.***Fusarium oxysporum* strain was isolated from samples that present fungal infection. The infected samples were placed in a PDA medium to allow fungal growth. After a small portion of the growing mycelium is reseeded in a box with fresh PDA medium and allowed to grow at 28 ${}^{\circ }$C or room temperature for 5 to 7 days. Analysis of conidia production is conducted by optical microscopy. Conidia were concentrated in phosphate buffer. Fungal concentration was determined by UV-Vis spectroscopy ($1\times 10^{7}$ CFU/mL).

**Drip dilution test.** For the drip dilution test, six decimal dilutions were made in sterile distilled water (by duplicate) to evaluate the fungal growth in each dilution and to demonstrate fungal bioavailability under the experimental conditions. The initial concentration of fungal cells was $1\times 10^{7}$ CFU/mL. From each dilution, an aliquot of 10 μL was laid on a Petri dish with SDA. The drops were allowed to dry to avoid movement displacement. They were left in an incubator at 28 ${}^{\circ }$C for 48 h (*C. albicans*) or 72 h (*S. schenckii*).

**Interaction test.** 100 μL are taken from the stock fungal suspension, placed in Eppendorf tubes and diluted to 1 mL. The dilution of the fungal suspension is $1\times 10^{6}$ CFU/mL. The interaction of fungi with NMs at different concentrations was performed then according to Table 1. Each experimental condition was evaluated by duplicate. The interaction was carried out for 5 h in a water bath at 37 ${}^{\circ }$C. The tubes were removed next from the water bath, then 100 μL were taken from each Eppendorf tube and placed on SDA (two boxes per tube), dispersed with boiling pearls and left in an incubator for 48 h (*C. albicans*) and 72 h (*S. scenckii*) for their subsequent analysis or counting at 28 ${}^{\circ }$C. The fungicidal activity of the materials was checked by determining the MIC (minimal inhibitory concentration) and MFC (minimal fungicidal concentration). The MIC was defined as the lowest concentration of the composite that significantly (visually) inhibited fungal growth with respect to CFU. The MFC was defined as the lowest concentration of the composite that completely inhibited fungal growth.

**Evaluation of the interaction of*****S. schenckii*****and*****C. albicans*****with CuI by Atomic Force Microscopy (AFM).** The analysis of the interactions of CuI@AG and CuI@Ch with the fungi was carried out by Atomic force microscopy. A 10 μL sample of the different treatments specified in the paragraph *Interaction test* was withdrawn after the exposure time was finished. The sample was deposited on a mica substrate and fixed with absolute ethanol. The samples were analyzed using a Dimension edge with a scan assyst microscope (Bruker) on tapping mode. Topography, phase, and amplitude images were collected using tapping mode in the air at room temperature. OTESPA-R3 probes (K: 26 N/m, f${}_{0}$: 300 kHz) were used for the analysis.

**Evaluation of the biocompatibility of CuI colloidal materials.** In vitro toxicity studies were carried out using human whole blood. Heparin-stabilized human blood was freshly collected. Physiological saline solution (PSS) was added to test tubes (10 mL of PSS in each tube). Different amounts of CuI@AG were added to each tube according to Table 2. A 100 μL sample of whole blood was added to each tube. All samples were prepared in triplicate, and the suspension was briefly vortexed before putting the samples in a water bath (37 ${}^{\circ }$C) for 5 h. The mixtures were centrifuged next at 3500 rpm for 5 min. The amount of hemoglobin was determined by UV–Vis spectroscopy (Thermo Scientific, Helios Omega UV-Vis) and measured with the reference wavelength of 525 nm.

## 3. Results and Discussions

### 3.1. Synthesis of CuI Nanostructured Materials

**Figure 2:**
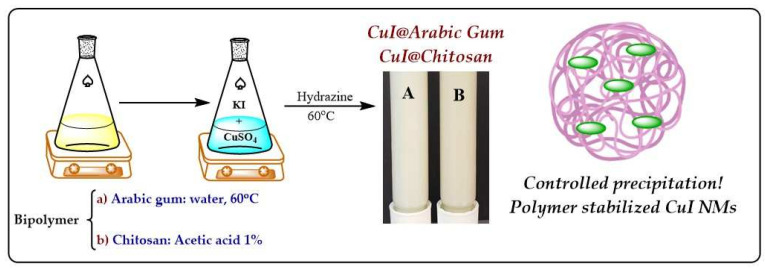
Synthesis of CuI nano-structured materials by a controlled precipitation method followed of reduction. Biopolymer (Arabic gum and chitosan were employed to increase colloidal stability). The materials (powders and colloids) exhibit a white color, characteristic of Cu(I) materials.

CuI powdered materials show a fine powder consistency and light creamy color. A high yield of the synthesis reaction was observed (>90%). White homogeneous colloidal suspensions of CuI were also fabricated (Figure 2). The colloids show stability for about two weeks. There are no significant differences in the appearance of the colloidal suspensions, concerning the polymer employed. The absence of greenish-blue tonalities confirms the reduction of Cu${}^{2+}$to Cu${}^{1+}$ species.

### 3.2. Characterization of CuI Nanostructured Materials

**Figure 3:**
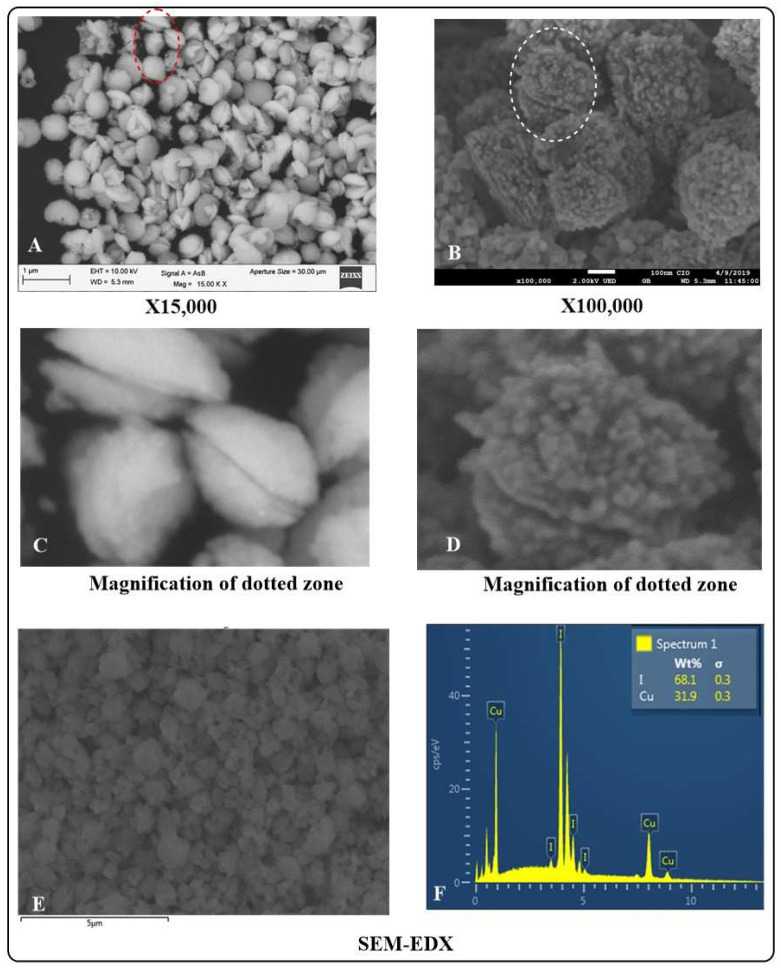
SEM-EDX analysis of CuI powdered nano-structured materials. (**A**) low magnification SEM micrograph of CuI powdered material, the shell like morphology of the materials is easily seen. (**B**) high magnification SEM micrograph of CuI powdered material, illustrating the ordered nanostructures that compose the 3D shell microstructures. (**C**) magnification of dotted zone in A. (**D**) magnification of dotted zone in B. (**E**) SEM micrograph for EDX analysis. (**F**) EDX analysis of CuI powdered materials.

**SEM-EDX.**Figure 3 shows the SEM characterization of CuI nanostructured materials. At low magnifications, the materials exhibit a shell-like morphology (Figure 3A,C). Figure 3C clearly shows the 3D shell-like architectures with controlled internal microstructures. Figure 3B,D show the materials at higher magnifications. The materials are composed of smaller particles that are orderly aggregated in 3D shell structures. SEM-EDX analysis (Figure 3E,F) reveals the composition and purity of the materials. The weight percentages of the elements are in agreement with a CuI formulation (33.4% Cu and 66.6% I). Figure S1 (Supplementary information) shows a representative micrograph of CuI with the corresponding EDX elemental mapping for copper and iodine. Only Cu and I are present and well dispersed in the CuI lattice, demonstrating the successful preparation of the material.

**Figure 4:**
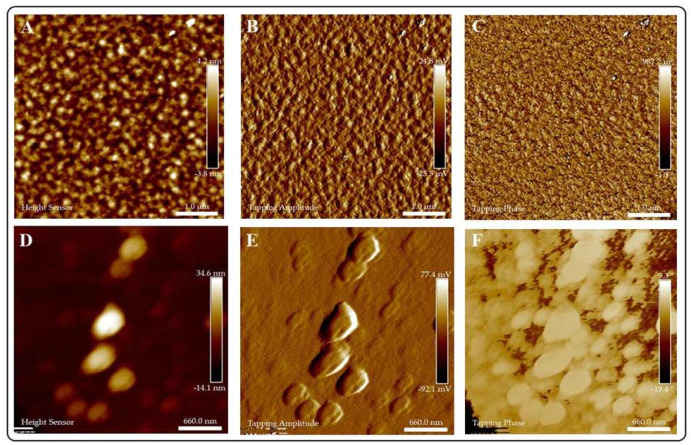
AFM analysis of Arabic gum (AG) and CuI@AG films. (**A**–**C**) Arabic gum thin films; height, amplitude and phase analysis, respectively. (**D**–**F**) CuI@AG thin films; height, amplitude and phase analysis, respectively.

**AFM.**Figure 4 portrays the AFM analyses of AG and CuI@AG thin films. Figure 4A–C show the morphology of AG films. The polymeric films (AG) are very thin (≈4 nm) and exhibit a uniform structure and composition. In comparison, CuI@AG films are thicker (≈30 nm) due to the insertion of CuI NMs in the polymeric matrix (Figure 4D–F). AFM analysis confirms the shell morphology of the CuI materials observed with SEM analysis. Tapping phase analysis (Figure 4C) of AG demonstrates the presence of pure polymer, while the CuI@AG phase analysis (Figure 4F) denotes phase changes due to inorganic (CuI) and organic (polymer) composition [21].

**Figure 5:**
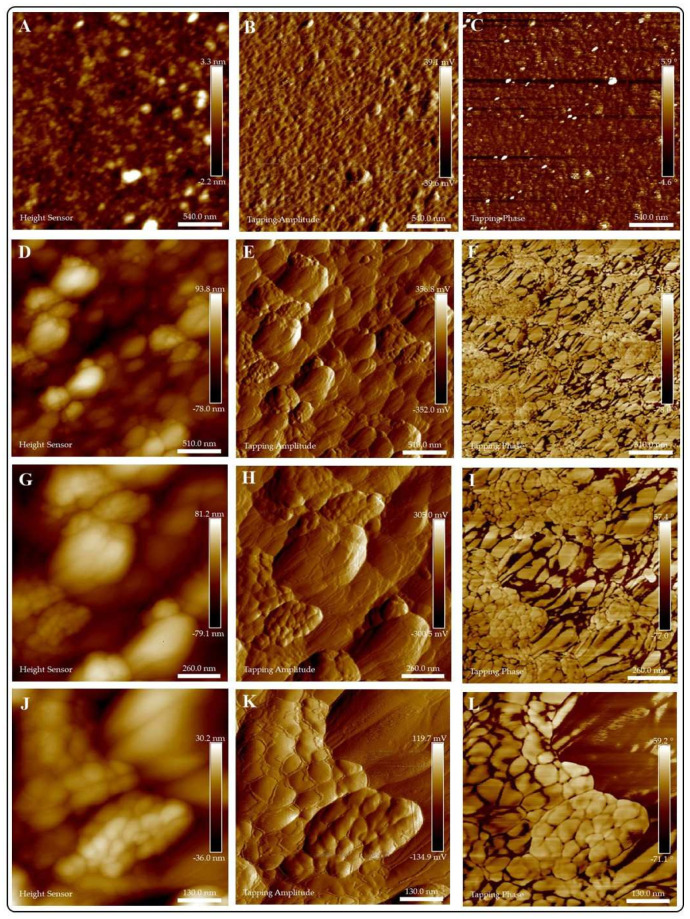
AFM analysis of Chitosan (Ch) and CuI@Ch films. (**A**–**C**) Chitosan thin films; Height, amplitude and phase analysis respectively. (**D**–**L**) CuI@Ch composite films; height, amplitude and phase analysis at different magnifications.

AFM analyses of chitosan and CuI@Ch films are presented in Figure 5. Two- dimensional topography analysis of chitosan is shown in Figure 5A, illustrating its smooth surface and homogeneity. Figure 5D–L display the height, amplitude, and phase images of CuI@Ch composites at increasing magnifications. The AFM images at low magnifications reveal the presence of uniformly distributed CuI nanostructured materials with shell-like morphology. At higher magnification, a detailed inspection of the composite nanostructure denotes the presence of small (≈ 50 nm) ordered particles with a hierarchical 3D shell-like morphology.

In tapping mode, phase analysis is capable of imaging the nanostructure of blend or multiphase materials with high resolution of submicron length scales. We can observe evident differences in the phase images of chitosan films (Figure 5C) in comparison to CuI@Ch films. For Ch films, only one phase is observed, corresponding to the biopolymer, whereas for the composite, inorganic (CuI) and organic (polymer) phases are represented.

### 3.3. Evaluation of the Antifungal Activity of CuI

Following our methodology, different variables were evaluated to optimize the interaction of CuI materials and pathogenic fungi. Our first attempt involved the use of CuI powdered material at different doses (0.5, 2.5, and 12.5 mμg/mL). Although still a common practice, the use of powdered nanomaterials for biomedical applications is cumbersome, since stable colloidal suspensions in exposition media are difficult to be obtained (Figure S2). The evaluation of the antifungal activity of CuI against *S. schenckii* was hampered by the aggregation of CuI materials in phosphate buffer, decreasing the interactions of NPs and fungi and limiting the reproducibility of observed results.

**Figure 6:**
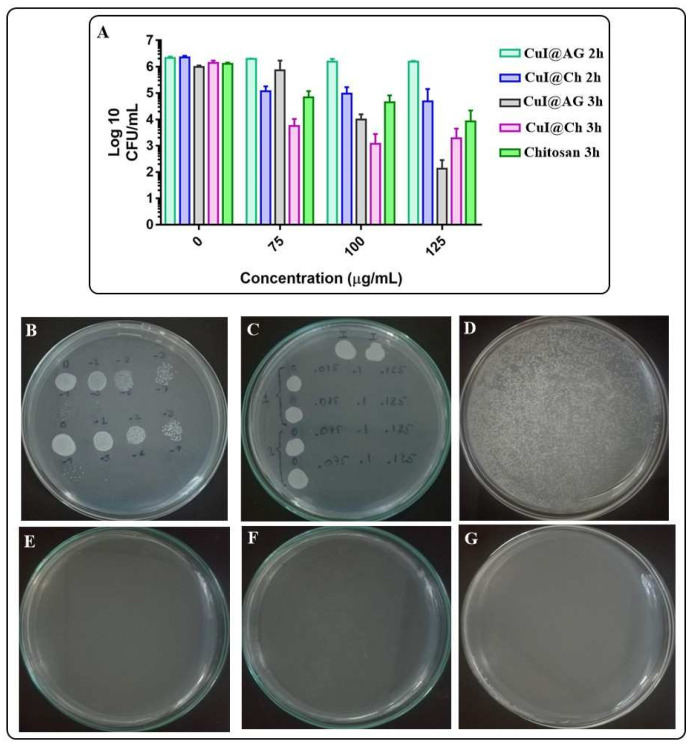
Evaluation of the antifungal activity of CuI@AG, CuI@Ch and chitosan to inhibit *C. albicans* growth. (**A**) Log reduction on the CFU/mL of *C. albicans* exposed to different amounts of CuI colloids or chitosan. (**B**) Viability of control group (decimal dilutions) under experimental conditions. (**C**) Evaluation of the fungicidal activity of CuI@AG against *C. albicans* (drop dilution test). (**D**–**G**) Enumeration of the CFU/mL of *C. albicans* exposed for 5 h to different amounts (0, 75, 100 and 125 μg/mL) of CuI NMs (spread plate procedure).

Because of the above results, stable CuI colloidal suspensions were prepared by using biopolymers as surfactants. Their antifungal activity was evaluated next at different concentrations of CuI colloids and times of exposition of pathogenic fungi. To avoid the aggregation of the NMs, their interactions with fungi were evaluated in distilled water. Initially, the following concentrations (75, 100, and 125 μg/mL) were evaluated at short exposition times (2 and 3 h). We observed good antifungal activity of the materials at all tested concentrations (Figure 6A). For instance, CuI@Ch shows antifungal activity with very high inhibition growth percentages (>90%). In addition, CuI@AG shows moderate activity after 2 h of exposition with an inhibition of 28% for the highest tested concentration. However, after 3 h of exposition, the activity of CuI@AG is very similar to CuI@Ch with inhibitions near 100%. Figure 6B shows that the fungi are viable (under the experimental conditions) in the growth controls at different serial decimal dilutions. Figure 6C illustrates the antifungal activity of the materials at different doses evaluated by the drop dilution method. Fungal growth is only registered for control groups. Figure 6D–G and Figure S4 show that longer exposition time (5 h) of fungi to these materials concentrations, result in complete growth inhibition. Figure S4 illustrates a slightly decreased antifungal activity of non-freshly prepared CuI@Ch colloidal suspension.

Freshly prepared CuI@Ch shows an enhanced antifungal activity in comparison to CuI@AG due to the synergic action of CuI and the polymer. However, its improved antifungal activity varies with time (Figure S4). A recent report remarks the importance to study the stability of biopolymers in different exposure media [22]. Accordingly, under experimental conditions, CuI@Ch tend to form aggregates, decreasing antifungal activity. Lixiviation of copper ions is one of the proposed mechanisms of CuI antifungal activity. For CuI@Ch composite, complex formation (Cu-NH${}_{2}$) results in decreased bioavailability of Cu ions or amino groups, the chemical species responsible for antifungal activity [23,24]. In addition, decreased solubility in the exposure media lowers the stability of CuI@Ch colloidal suspension. Earlier reports point out the role of the physicochemical properties of chitosan and its antifungal activity. For example, the high molecular weight chitosan shows a better antifungal activity. Moreover, the susceptibility of the fungi specie is an important consideration [25].

Figure S5 shows the results of the antifungal activity of Cu NPs. Figure S5A illustrates the presence of fungi after their interaction with Cu NPs treatment. In contrast, CuI materials inhibit the growth of the fungi under the same experimental conditions. Figure S6 illustrates the viability of the fungal cells under experimental conditions (top row Figure S6A). It is evident the effect of Cu NPs treatment at different doses. CuI composites achieve more than 90% growth inhibition. For Cu NPs, lower efficiencies are attained under the same experimental conditions (Figure S6B). Accordingly, a recent study discusses the effect of Cu and CuO NPs to inhibit *Colletotrichum gloeoesporioides*. Cu and CuO NPs affect fungal growth at high doses (500 mμg/mL) and long exposure times [26]. Cu NPs exert morphology-dependent antifungal activity. For example, the sharp edges of marigold-like petal nanostructures injure the cellular wall and membrane, and cause the death of the yeast (*C. albicans*) [27].

Our results indicate that the conidia of *S. schenckii* are more susceptible than the yeast of *C. albicans* to CuI treatment. For example, after 5 h of the exposition of these fungi to CuI@Ch, total growth inhibition is observed at all evaluated concentrations. Also, if the time of exposition is reduced (2 h), then the fungal growth inhibition is achieved at a concentration of 75 μg/mL of CuI@Ch. CuI@AG shows similar behavior for the treatment of *S. schenckii*. For example, after 5 h of the exposition of *S. schenckii* to CuI@AG composite, the minimum inhibitory concentration (MIC) is 12.5 μg/mL, whereas the minimum fungicidal concentration (MFC) is 25 μg/mL. At shorter exposition times (2 h), 125 μg/mL of CuI@AG composite must be used to inhibit the fungal growth (Figures S7 and S8). To our knowledge, there are not many reports regarding the development of effective and specific compounds for the treatment of *S. schenckii* infections. Potassium iodide, azoles, and amphotericin B are among the very few options for fungal infection treatment. However, these treatments result in side deleterious effects. Except for KI, the cost of the treatment is elevated, a barrier for general application in developing countries [28]. In this work, we demonstrate the effective antifungal activity of CuI materials at low doses. Their affordable cost and biocompatibility suit them as strong candidates for the development of new broad-spectrum antifungal agents.

As previously discussed, *C. albicans* is more resistant to CuI treatment. For example, after the exposition of these pathogenic fungi to CuI@AG for 5 h, fungal growth is observed at the lower evaluated doses (12.5 and 25 μg/mL). However, good inhibition activity is reached, since the fungal growth in the control group is abundant, impeding the total (CFU), direct count. On the other hand, after treatment with 12.5 μg/mL, only 50 CFU are observed. Also, treatment with 25 μg/mL decreases the growth to 5 CFU. CuI@Ch colloids exhibit the same behavior discussed for *S. schenckii*. The materials inhibit the growth of *C. albicans* at all evaluated concentrations after 5 h of exposition. However, variable effectiveness is also observed. The variability might also be a consequence of the instability of the polymer at the exposure media (pH and ionic strength).

Figure S9 shows the preliminary results on the fungicidal activity of CuI@AG against *Fusarium oxysporum*. Our results indicate fungal growth inhibition at high doses (75 μg/mL) and 20 h exposure. Future studies aim to optimize the different interaction variables to increase antifungal efficiency. Previous studies discuss the advantages of the use of NMs for the control of fungal plant diseases. Carbon, silver, silica, non-metal oxides, polymer composites and aluminosilicates show efficient activity to promote plant growth and to inhibit plant pathogens [29]. CuI@AG is a potent candidate for the development of fungicidal agents for crop protection. Also, NMs are efficient for the treatment of superficial fungal and yeast infections. Among the therapeutic agents, Ag, CuO, polymers and ZnO NPs have been investigated. NPs can also be used as antifungal carriers for the treatment of superficial fungal infections (liposomes, nanofibrous) [30] (Chapters 5, 6). From all the above, it is evident that nanotechnology offers numerous strategies to overcome the challenges that currently face the health sector.

**Figure 7:**
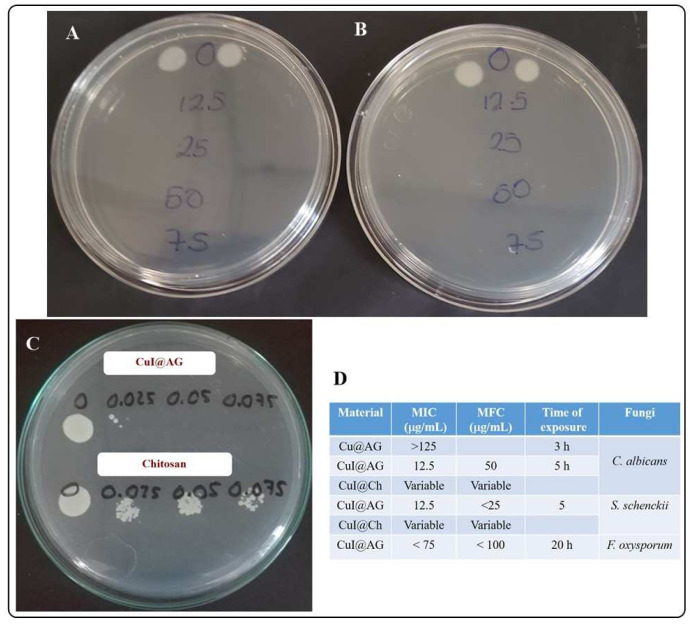
Summary of the antifungal activity of CuI NMs (5 h of exposure). (**A**) CuI@AG against *S. schenckii*. (**B**) CuI@Ch against *S. schenckii*. (**C**) CuI@AG and chitosan against *C. albicans*. (**D**) MIC and MFC values.

In summary, CuI@AG shows the best antifungal activity among the different materials evaluated in this study (Cu@AG, Chitosan, CuI@Ch). Figure 7 summarizes the effective doses of the materials to inhibit the growth of pathogenic fungi. Figure 7B shows the efficiency of CuI@Ch as fungicidal at low doses. However, Figure S4 shows the variable behavior of this material. This behavior is due to its instability in the exposure medium. Figure 7C shows the enhanced antifungal activity of CuI@AG in comparison to chitosan. Although numerous reports remark the antifungal activity of this polymer, CuI@AG is more effective. Figure 7D resumes the MIC and MFC for the antifungals under study.

The materials presented in this study offer numerous advantages against some previously reported antifungal compounds (Table 3). For instance: (a) the compounds can be synthesized using a green route under ambient conditions; (b) the materials show excellent antifungal activity at low doses and short exposure times; (c) the materials exhibit different mechanisms to inhibit the growth of different pathogenic fungi; (d) the materials show biocompatibility at the doses that exhibit antifungal activity; (e) Low cost.

### 3.4. Evaluation of the Interaction of Fungi and CuI by AFM

Because of their physicochemical properties, NMs can display different mechanisms to destroy pathogenic microorganisms. Thus, numerous analytical techniques are applied to elucidate the antimicrobial activity of NMs. In this study, we use AFM to investigate the capacity of CuI NMs to inhibit the growth of pathogenic fungi. As previously discussed in this work, CuI NMs exhibit antifungal activity at very low doses. This capacity is not only due to their composition but also to the different and individual antifungal mechanisms shown by the elements present in the composite.

The analysis of the interaction of CuI and *Candida* by optical microscopy (Figure S10) shows the capacity of the materials to inhibit biofilm formation. The colony-forming ability of *C. albicans* decreases as the amount of CuI NMs increases. The inhibition of biofilm formation is a result of the high affinity between fungi cells and NMs. Our observations are supported by previous studies that discuss the effectiveness of iodine-containing polymers to inhibit the growth of biofilm-forming microorganisms. The materials avoid the adhesion of bacteria (*S. aureus*) or fungi (*C. albicans*) to surfaces, limiting biofilms formation [32].

**Figure 8:**
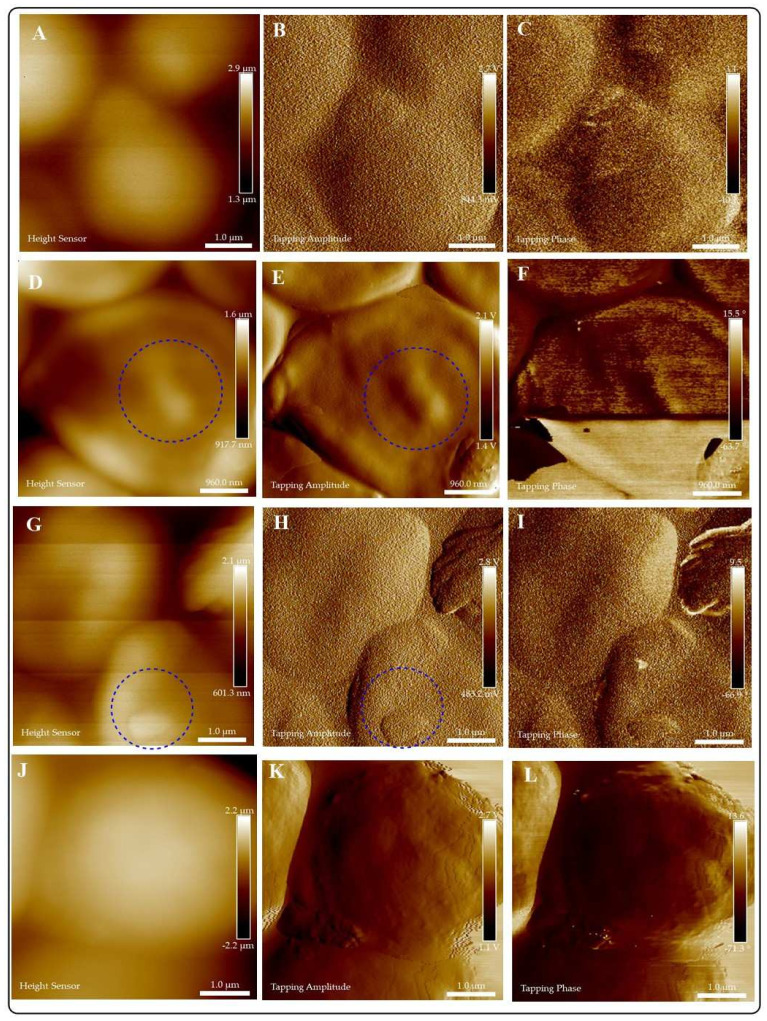
Evaluation of the exposition of *C. albicans* to different doses of CuI@AG for 1 h. Height, amplitude and phase images are presented for: Control group (**A**–**C**), and *C. albicans* exposed to different doses of CuI@AG: 75 μg/mL (**D**–**F**); 100 μg/mL (**G**–**I**); 125 μg/mL (**J**–**L**). Blue dotted circles indicate the presence of NMs disrupting fungal cells.

**Figure 9:**
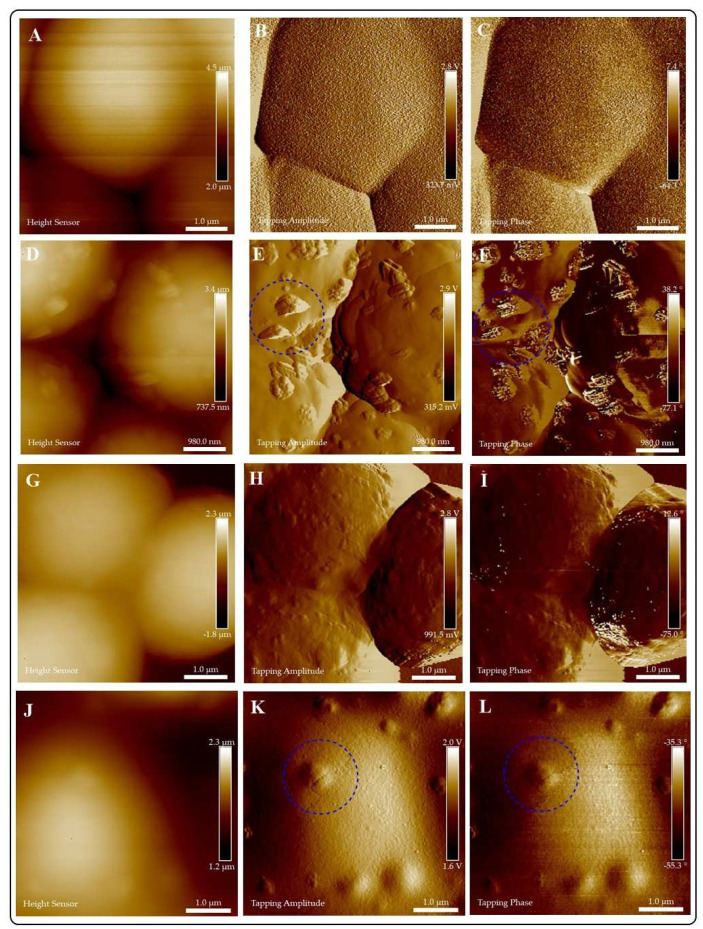
Evaluation of the exposition of *C. albicans* to different doses of CuI@Ch for 1 h. Height, amplitude and phase images are presented for: Control group (**A**–**C**), and *C. albicans* exposed to different doses of CuI@Ch: 75 μg/mL (**D**–**F**); 100 μg/mL (**G**–**I**); 125 μg/mL (**J**–**L**). Blue dotted circles indicate the presence of aggregated NMs on the surface (**D**–**F**) or penetrating the cells (**G**–**L**). Figures E and F denote the presence of nanomaterials on the surface of the fungi. In particular, face change in F corroborates the presence of the nanomaterials on the surface. On the other hand, K and μL illustrate the penetration of nanomaterials in fungal cells.

**Figure 10:**
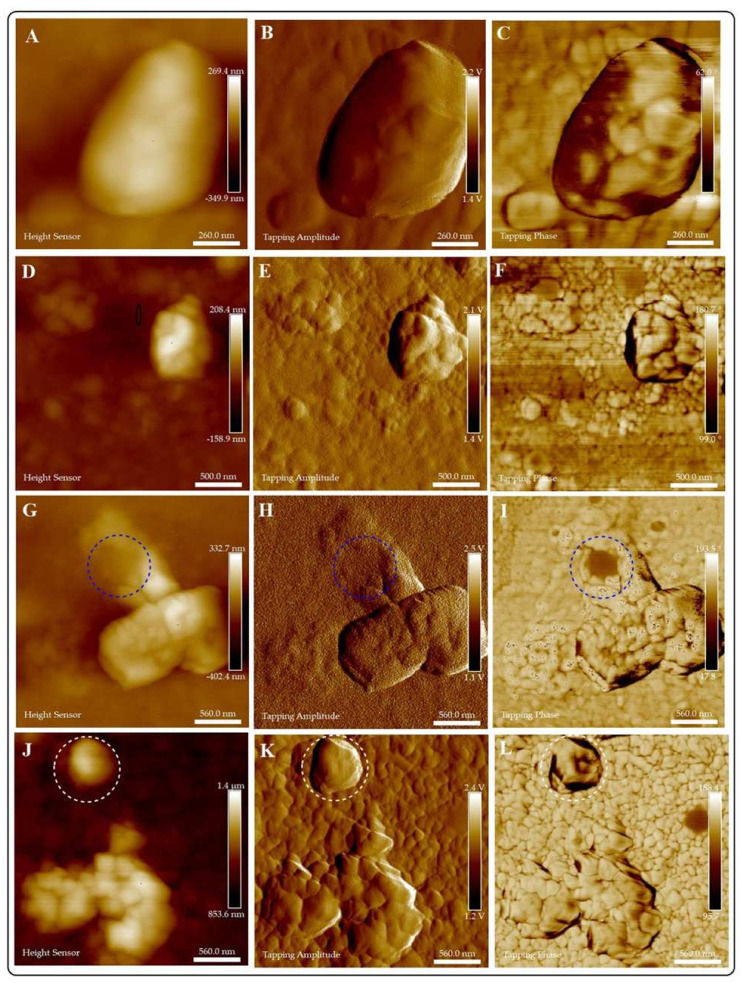
Evaluation of the exposition of *S. schenckii* to different doses of CuI@Ch for 1 h. Height, amplitude and phase images are presented for: Control group (**A**–**C**), and *S. schenckii* exposed to different doses of CuI@Ch: 75 μg/mL (**D**–**F**); 100 μg/mL (**G**–**I**); 125 μg/mL (**J**–**L**). Blue dotted circles indicate the penetration of the material on the fungal cell and exposition of its components. White dotted circles indicate morphological changes in *S. schenckii* due to the almost complete fungal cell coverage of the composites.

AFM allows a closer examination of the interactions of CuI colloids and pathogenic fungi. There are no differences in the affinity exhibited by the colloids to adhere and penetrate the fungal cell (Figure 8, Figure 9 and Figure 10). Short interaction times (1 h) demonstrate the adherence of the materials to fungi cell membranes. We can also observe differences in the stability of the colloids in the exposition media. For example, CuI@AG materials do not agglomerate, facilitating their interaction with fungal cells. This close interaction (CuI@AG-*C. albicans*) results in cell damage at all evaluated concentrations, as represented in Figure 8. Contrarily, CuI-Ch materials form big aggregates at the surface of the *C. albicans* membrane (Figure 9). Their increased size due to agglomeration decreases their antifungal activity. As previously discussed, the low stability of the materials renders poor reproducibility. This observation supports our results of the antifungal activity of these materials evaluated by classical microbiology methods.

The AFM analysis of the morphological changes experimented by fungi after interacting with CuI materials indicates the penetration and disruption of the cells (Figure 8). Previous studies reported that the adherence of NMs to the membranes of microorganisms increases the lag stage of the bacterial growth period, extending the reproduction time of the microorganisms [37,38]. In accordance, in this study, the growth of pathogenic fungi was slower as a result of their exposure to CuI composites.

Kim et al. [31] reported that as a result of the interactions of *C. albicans* and nano-Ag, the fungi membranes exhibit significant changes which were manifested by the formation of “pits” on their surfaces, followed by pore development and cell death [31]. Our results indicate similar findings, the formation of a pit on the surface of *C. albicans* due to CuI penetration (Figure 8 and Figure S11). Following the adherence of NMs to the membranes, different mechanisms of antifungal activity become active. For instance, the adhesion of CuI@Ch to fungal cell results in increased permeability of fungal membrane, then leakage of cellular contents, followed of cell death [25].

To our knowledge, there are not many reports regarding the antifungal activity of iodine based materials [32,39]. For example, the preparation of PVP-Iodine liposome hydrogels and their capacity to inhibit *C. albicans* growth was reported [33]. Human keratinocytes infected with *C. albicans* underwent antifungal treatment (PVP-iodine liposome hydrogels and commercially available Betaisodona). The iodine-based treatment results in epithelial alterations of the keratinocytes. Additionally, PVP-iodine liposome hydrogels show a multi-step antifungal mechanism. First, the adherence of the material to the cell wall of *C. albicans*, followed by membrane damage and adsorption of the active agent into the fungal cell. Our results illustrate similar findings. However, lower doses and exposure times are required.

The alteration on the morphology of *S. schenckii* due to interactions with CuI were evaluated by AFM. The results are presented in Figure 10 and S12. The high affinity of CuI colloidal materials to the *S. schenckii* cell surface results in the total coverage of fungal cells at all evaluated concentrations. AFM images show the typical morphological characteristics of these fungi by observation of the control group, demonstrating its bioavailability under test conditions (Figure 10A–C). CuI@Ch firmly adheres to fungal cells, causing damage at short exposition times (Figure 10G–L). As previously discussed by *C. albicans*, CuI@Ch forms bigger aggregates in the exposition media. Treatment of *S. schenckii* with doses of 100 and 125 μg/mL (for one hour) results in damage to fungal cells.

Figure 10G,H illustrate the formation of a cavity in *S. schenckii* (dotted blue circles). This asseveration is supported by tapping phase analysis (Figure 10I), which highlights the location of interfaces within the sample. Phase contrast is evident in the circled region, indicating the interface between NMs (light zones) and fungi (dark zone). The penetration of the materials disrupts the membrane exposing fungi cellular components. Figure 10J,K illustrate the changes in the morphology (dotted white circles) of *S. schenckii* due to interaction with the highest dose of composites. Figure 10I confirms the presence of morphologically modified fungi due to the almost complete coverage of fungi surface.

By AFM studies, we demonstrate that the highest susceptibility of *S. schenckii* to CuI materials is due to closer interactions of the materials and fungal cells. There are evident differences in the size of the fungal cells evaluated in this study (conidia of *S. schenckii* vs. *C. albicans* yeast), a variable that might influence the reported results. There are no significant differences in the activity of CuI@Ch or CuI@AG. For practical applications, CuI@AG is more suitable. Because of its abundance, low-cost, high hydrophilicity, and low toxicity. These properties suit it as an excellent candidate for the development of composites for biomedical and environmental applications.

### 3.5. Evaluation of the Bio-Compatibility of CuI@AG

**Figure 11:**
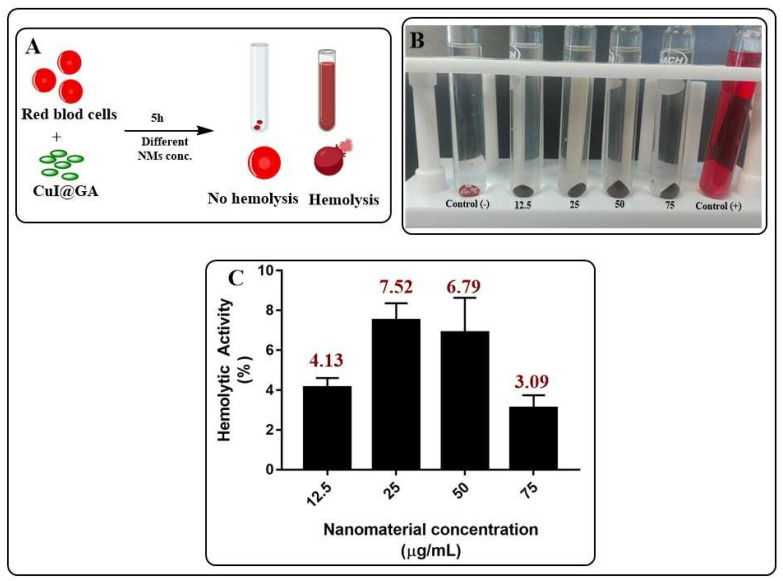
Evaluation of the hemolitic activity of CuI@AG. (**A**) Schematic representation of the interaction of red blood cells and CuI@AG. (**B**,**C**) Qualitative and quantitative evaluation of the hemolitic activity of CuI@AG at different doses.

Due to the membrane-damaging effects of CuI@AG composites in pathogenic fungi, we studied the outcomes of the interaction of composites and human red blood cells (RBCs). An *in vitro* toxicity assay to evaluate the hemolytic activity of the composites at different doses was conducted (Figure 11A). As shown in Figure 11B,C, the composites exhibited little hemolytic activity at doses where the effective antifungal activity occurs. Side effects in host cells (RBCs) result in their exposition to composite materials at 12.5 μg/mL. We can also observe a decreased hemolytic activity at higher doses of the materials. This behavior might indicate the aggregation of the composite materials in the exposure media. The homo-aggregation of the materials decreases its surface area, thus its reactivity toward surrounding RBCs.

It is important to remark that the interactions of NMs and living cells are complex and depend on the surface properties of the NMs and the functional groups present in the different cell membranes [40,41]. Exposure media also influence the surface properties of NMs, thus also their bio-activity. For example, Ag NPs can inhibit the growth of some, but not all, fungal strains. Another example indicates that the preparation of ketoconazole-loaded chitosan–gellan gum nanoparticles are more effective against *Aspergillus niger* than unmodified NPs and ketoconazole alone [42,43]. Current findings are not contradictory: the differences arise from the lack of uniformity in the experimental conditions among research groups.

Also, numerous reports indicate increased biocompatibility of metallic NPs after polymer functionalization. In the present study, we use Arabic gum and chitosan as CuI coatings. It is important to point up that Arabic gum is negatively charged under experimental conditions, whereas chitosan has a positive charge. RBCs are also negatively charged, avoiding electrostatic interactions with Arabic gum coated CuI materials. The AFM study of the interactions of fungal cells and CuI@polymers shows the adsorption of the composites to the fungal cells. After adsorption, the NMs enter the fungal cell, causing its death. Fungal cells contain a cell wall that can serve as a reservoir for CuI accumulation. RBS lack a cell wall. Numerous reports indicate that lixiviation of ions is favorable after a direct interaction with biological entities. CuI@AG does not interact closely due to the electrostatic repulsion of negatively charged surfaces. Penetration of NMs into the cells is not favorable under such conditions.

Although in this manuscript we present the most relevant findings related to the antifungal activity of CuI materials, previous studies show that CuI@Ch become unstable in the exposure media (aggregation), decreasing their antifungal activity. We also observe that, due to their positive charge under physiological conditions, these materials exert hemolytic activity. Our results show the same trend during the antifungal or bio-compatibility evaluation. The activity of CuI@Ch NMs is dose and time-dependent due to their instability in the exposure media. Current studies of our research group aim to improve CuI@Ch stability.

Finally, in a previous work, Pd@Ag nanosheets were studied as potential alternatives to develop efficient antifungal agents. The materials exhibit enhanced antifungal activity and biocompatibility at low doses [35]. The composites (CuI@AG) under study in the present manuscript also exhibits enhanced antifungal activity and biocompatibility in human red blood cells. CuI@AG composite materials can be a better option for large scale applications due to their lower cost and simple fabrication.

## 4. Conclusions

In work work, CuI@AG and CuI@Ch composites were synthesized using a facile and reproducible technique. The materials exhibit a 3D shell ordered structure that might suit them for numerous applications. We demonstrated antifungal activity at low doses and short exposure times. We also demonstrate the enhanced activity of CuI by comparing it with Cu NPs antifungal activity. The CuI NMs are bio-compatible with RBCs at the doses required to exert antifungal activity. The materials display excellent antifungal activity against biofilm-forming pathogenic fungi. The materials are also efficient to inhibit the growth of filamentous fungi (*Fusarium oxysporum*). To our knowledge, this is one of the first studies that demonstrate the efficacy of nano-antifungals to inhibit *S. schenckii* growth. Future studies will investigate the activity of the materials using *S. schenkii* yeasts. Commercially available fungal therapies show limited effectiveness due to their reduced fungal cell penetration and the development of drug-resistant strains. In this work, we demonstrate the multistep antifungal mechanism of CuI composites. Multiple studies have shown the strong antimicrobial effects of single metallic NPs on several species of microorganisms. However, some NPs are effective against certain species but have little or no effect on others. Aiming to formulate a broad-spectrum antifungal, Cu, I, and chitosan integrate the present formulation. The combination of different antifungal agents renders enhanced activity at low doses. This favors its biocompatibility with host cells and reduce the capacity of the pathogen for resistance development. It is important to remark that the synthesis of CuI@AG develops within minutes under ambient conditions. The materials are efficient, bio-compatible, broad-spectrum fungicidal agents, and of low cost. Due to all the above considerations, CuI@bio-polymer composites turn out to be potent candidates to develop efficient antifungal agents for biomedical or environmental applications.

## Figures and Tables

**Table 1:** Determination of the MIC and MFC of CuI@polymer composites. The exposure of pathogenic fungi was evaluated at different composites concentrations.

CuI (μg/mL)	Distilled Water (μL)	CuI@AG Colloidal Suspension (μL)
0	900	0
12.5	896	4
25	892	8
50	884	16
75	876	24
100	868	32
125	860	40

**Table 2:** Determination of the biocompatibility of CuI@polymer composites. The exposure of RBCs was evaluated at different composites concentrations.

Tube	Concentration (μg/mL)	CuI (g)	PSS (mL)	Distilled Water (mL)	Blood (μL)
Control −	0	–	10	0	100
1	12.5	40	9.96	0	100
2	25	80	9.92	0	100
3	50	160	9.84	0	100
4	75	240	9.76	0	100
Control +	–	–	–	10	100

**Table 3:** Comparison of the properties of non-conventional antifungal agents.

Material	Advantages	Disadvantages	Reference
CuI NPs	Broad-spectrum antibacterial and antifungal agent.	Long exposition times (24 h). The biocompatibility of the materials was not evaluated. High doses (100–150 μg/mL).	[7]
Ag NPs	Good antifungal activity at low doses (2 μg/mL)	Low fungal cell density ($1\times 10^{4}$/mL), Synthesis protocol indicates AgCl formation.	[31]
Chitosan NPs	Broad-spectrum antifungal activity	High doses	[25]
Cuprous iodide complexes with phenantrolines	Good antifungal and antibactericl activity at low doses (1.25–2.5 μg/mL)	Cumbersome synthetic procedure. Long exposition times (24 h). Elevated cost.	[23]
Farnesol-containing chitosan NPs	Reduces the pathogenicity of C. albicans in a murine model. Fungicidal activity at low doses	Long exposure times (48 h)	[3]
PVP-I, PEO/PVP-I complexes	Antibacterial and antifungal activity. Short time of exposure.	High doses.	[32]
PVP-I liposome hydrogel	In vitro model of oral candidosis	Irritation in epithelium. External use (ointment)	[33]
Schiff base zinc complexes with thiocyanate an iodide	Antibacterial and antifungal activity	Soluble in organic solvents, High MIC values (256 μg/mL). Expensive.	[34]
Pd@Ag Nanosheets	Broad-spectrum antifungal agent. Biocompatibility with Red Blood Cells.	Long exposure times (24 to 72 h), cumbersome synthetic procedure, expensive.	[35]
Co, CuO NPs	Antifungal activity against devastating plant fungi pathogen	High doses (500 mμg/mL), long exposure times	[26]
Cu NPs	Inhibition on the growth of fluconazole resistant *C. albicans*	High doses	[36]

## Data Availability

The data presented in this study are available in insert article or supplementary material here.

## Supplementary Materials

The following are available online at https://www.mdpi.com/2309-608X/7/2/158/s1, Figure S1. SEM-EDX analysis of CuI nanostructured materials. (A) SEM micrograph of powdered CuI NMs. (B) EDX analysis highlighting copper content. (C) EDX analysis highlighting iodide content. The mass (wt) and atomic (at) percentages of the elements confirm the formation of CuI materials; Figure S2. SEM micrographs illustrating the behavior of powdered CuI nanostructured materials at different doses in phosphate buffered medium in the presence (A*–C*) and absence (A–C) of *S. schenckii*; Figure S3. UV-Vis characterization of Cu@AG colloidal suspension.The spectra shows the characteristic plasmon of Cu NPs; Figure S4. Evaluation of the antifungal activity (*C. albicans*) of CuI@AG (A), chitosan (B) and CuI@Ch (C) at different doses; Figure S5. Evaluation of the antifungal activity (*C. albicans*) of Cu@AG (A), growth from dilution (10${}^{-3}$) (B) at different doses; Figure S6. Evaluation of the antifungal activity of Cu@AG to inhibit C.albicans growth. (A) Viability of control group (decimal dilutions) under experimental conditions (Top row). Evaluation of the fungicidal activity of Cu@AG against *C. albicans* (drop dilution test) at differen NPs concentrations. (B) Log reduction on the CFU/mL of *C. albicans* exposed to different amounts of Cu@AG; Figure S7. Evaluation of the antifungal activity (*S. schenckii*) of CuI@AG at different concentrations and exposition of 5 h. (A) Fungal cell are viable in the growth controls at different serial decimal dilutions under experimental conditions. (B) Evaluation of the antifungal activity of CuI@AG by the drop dilution method. Fungal growth is only observed for growth control. (C–F) Evaluation of the antifungal activity of CuI@AG by the pour plate method. Fungal growth is only observed for growth control (C); Figure S8. Evaluation of the antifungal activity (*S. schenckii*) of CuI@Ch at different concentrations and exposition of 5 h. (A) Fungal cell are viable in the growth controls at different serial decimal dilutions under experimental conditions. (B) Evaluation of the antifungal activity of CuI@Ch by the drop dilution method. Fungal growth is only observed for growth control. (C–F) Evaluation of the antifungal activity of CuI@Ch by the pour plate method. Fungal growth is only observed for growth control (C); Figure S9. Evaluation of the antifungal activity (*F. oxysporumi*) of CuI@AG at different concentrations and exposition of 20 h. (A) Fungal cell are viable in the growth controls at different serial decimal dilutions under experimental conditions. (B–D) Evaluation of the antifungal activity of CuI@AG at different doses by the drop dilution method. Fungal growth is only observed for the fungi exposed to 75 μg/mL of NMs; Figure S10. Optical microscopy examination of CuI-C. albicans interaction at diferent CuI doses. (A) Big aggregates of *C. albicans* are observed for control group. (B–D) The aggregates decrease as the concentration of NMs increases (50, 75 and 100 μg/mL respectively); Figure S11. AFM analysis of the morphological changes in *C. albicans* due to exposure to CuI@AG (50 μg/mL × 1 h). Formation of a pit in fungal cell is observed due to NMs penetration (red dotted circles). A,B,C are the low-magnification images, whereas D,E,F are the high magnification images; Figure S12. AFM analysis of the morphological changes in S. schenckii due to exposure to CuI@Ch (50 μg/mL × 5 h). (A–C) Control group. The fungi maintain their typical morphology under test conditions, demonstrating the bioavailability of the MOs at the end of the experiment. (D–I) The NMs interact closely with fungal cells resulting in their destruction.

## Sample Availability

Samples of all the compounds are available from the corresponding author.
