# Supplementary material for: Development of Nano-Antifungal Therapy for Systemic and Endemic Mycoses
Source: J Fungi (Basel). 2021 Feb 23;7(2):158. doi: 10.3390/jof7020158 (PMC7926374; doi:10.3390/jof7020158)

Supplementary Information.

Development of Nano-Antifungal therapy for systemic and endemic mycoses

Jorge H. Martinez-Montelongo[1], Iliana E. Medina-Ramirez[1,*], Yolanda Romo-Lozano[2], Antonio Gonzalez-Gutierrez[1], Jorge E. Macías-Díaz[3,4]

1. Department of Chemistry, Universidad Autónoma de Aguascalientes. Av. Universidad 940, Aguascalientes, Ags. México.
2. Department of Microbiology, Universidad Autónoma de Aguascalientes.
3. Department of Mathematics, School of Digital Technologies, Tallinn University, Narva Rd. 25, 10120 Tallinn, Estonia.
4. Department of Mathematics and Physics, Universidad Autónoma de Aguascalientes.

[*]Author for correspondence. Email: iemedina@correo.uaa.mx

Figure S1. SEM-EDX analysis of CuI nanostructured materials. A) SEM micrograph of powdered CuI NMs. B) EDX analysis highlighting copper content. C) EDX analysis highlighting iodide content. The mass (wt) and atomic (at) percentages of the elements confirm the formation of CuI materials.

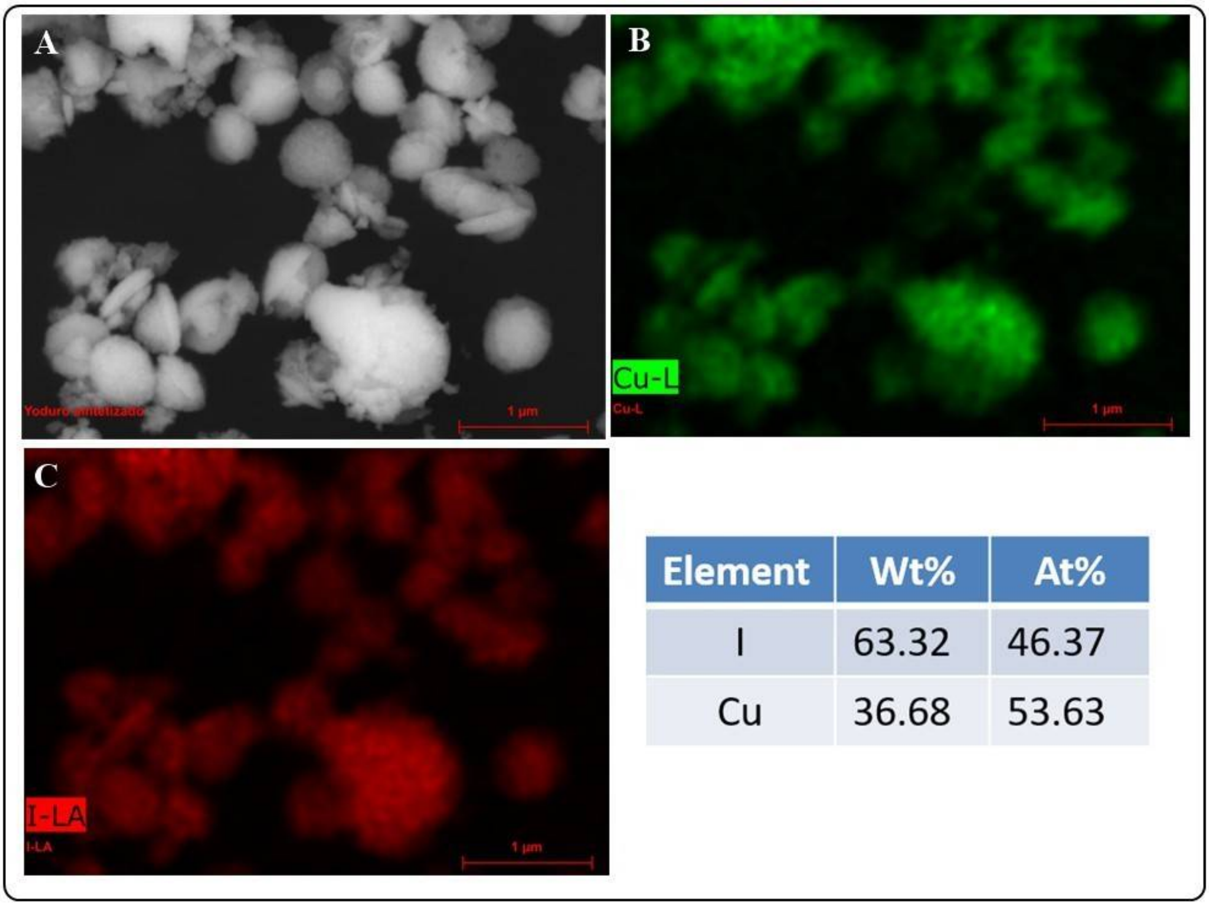

Element	Wt%	At%
I	63.32	46.37
Cu	36.68	53.63

Figure S2. SEM micrographs illustrating the behavior of powdered CuI nanostructured materials at different doses in phosphate buffered medium in the presence (A*–C*) and absence (A–C) of *S.schenckii.*

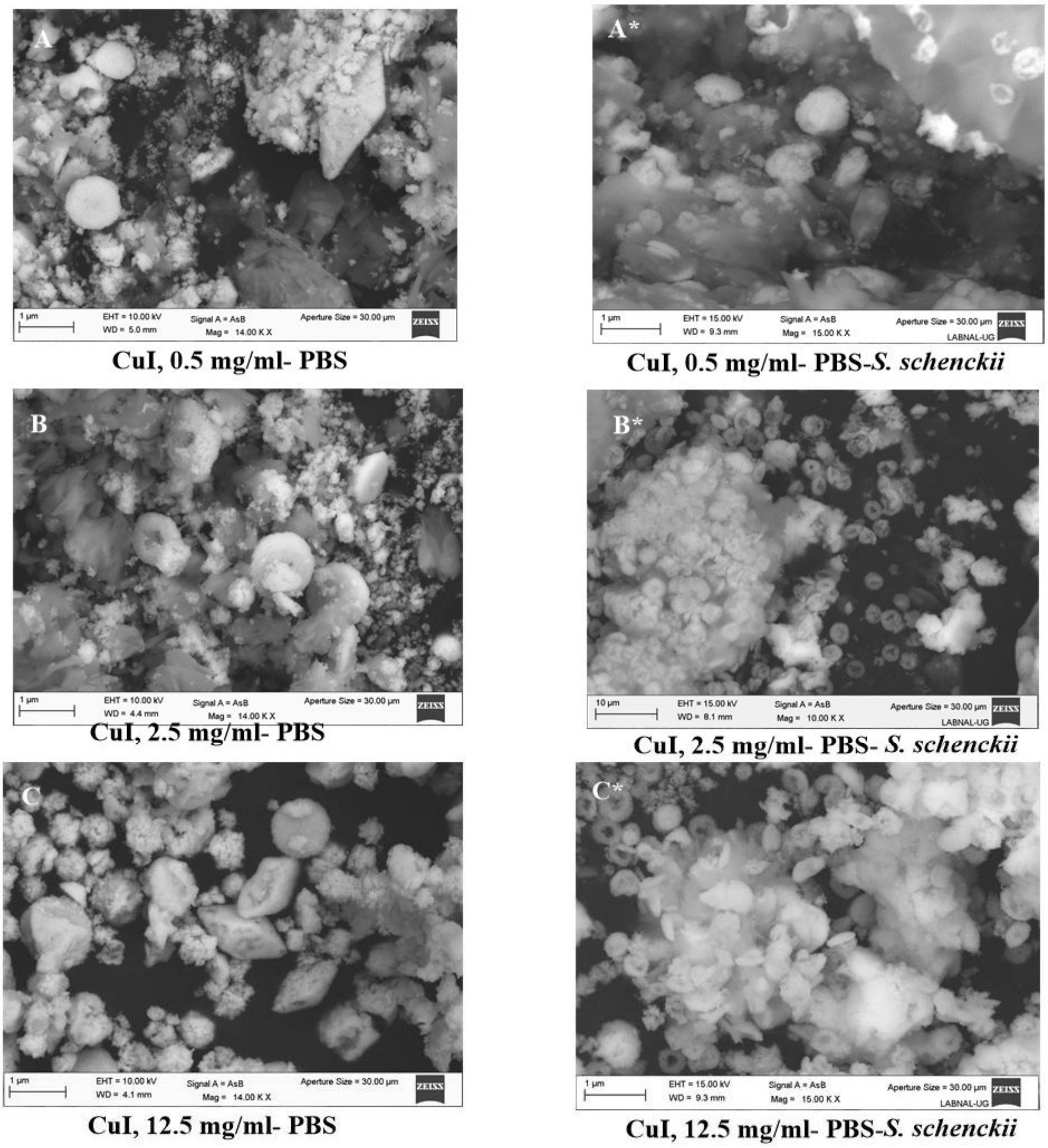

G Element	Atomic percentage composition					
	A	B	C	A*	B*	C*
Cu	25.68	27.35	9.80	58.5	N.A.	28.01
I	9.00	16.57	1.13	13.37	N.A.	19.89
O	52.47	46.74	73.92	4.13	N.A.	16.05
P	11.76	8.20	11.78	0.88	N.A.	0.44
K	1.09	1.13	3.37	0.97	N.A.	-
C	-	-	-	22.51	N.A.	35.61

Figure S3. UV-Vis characterization of Cu@AG colloidal suspension. The spectra shows the characteristic plasmon of Cu NPs.

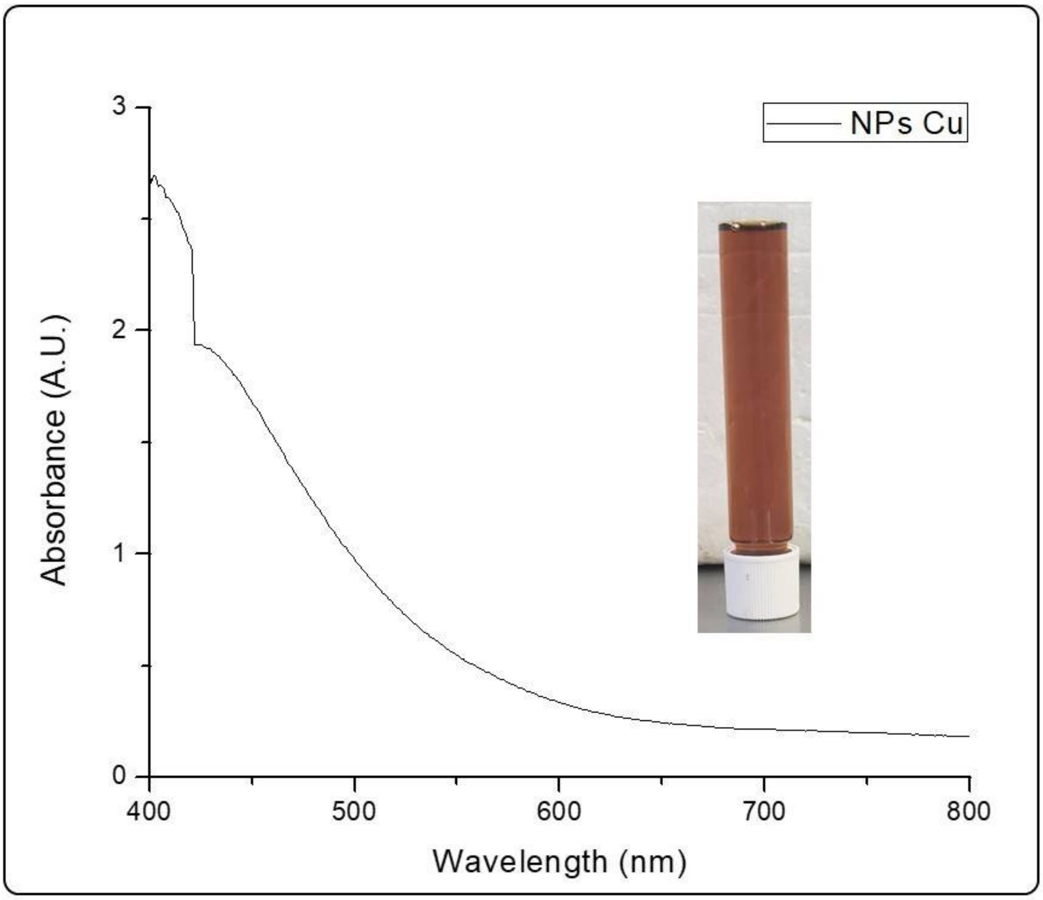

Figure S4. Evaluation of the antifungal activity (*C. albicans*) of CuI@AG (A), chitosan (B) and CuI@Ch (C) at different doses.

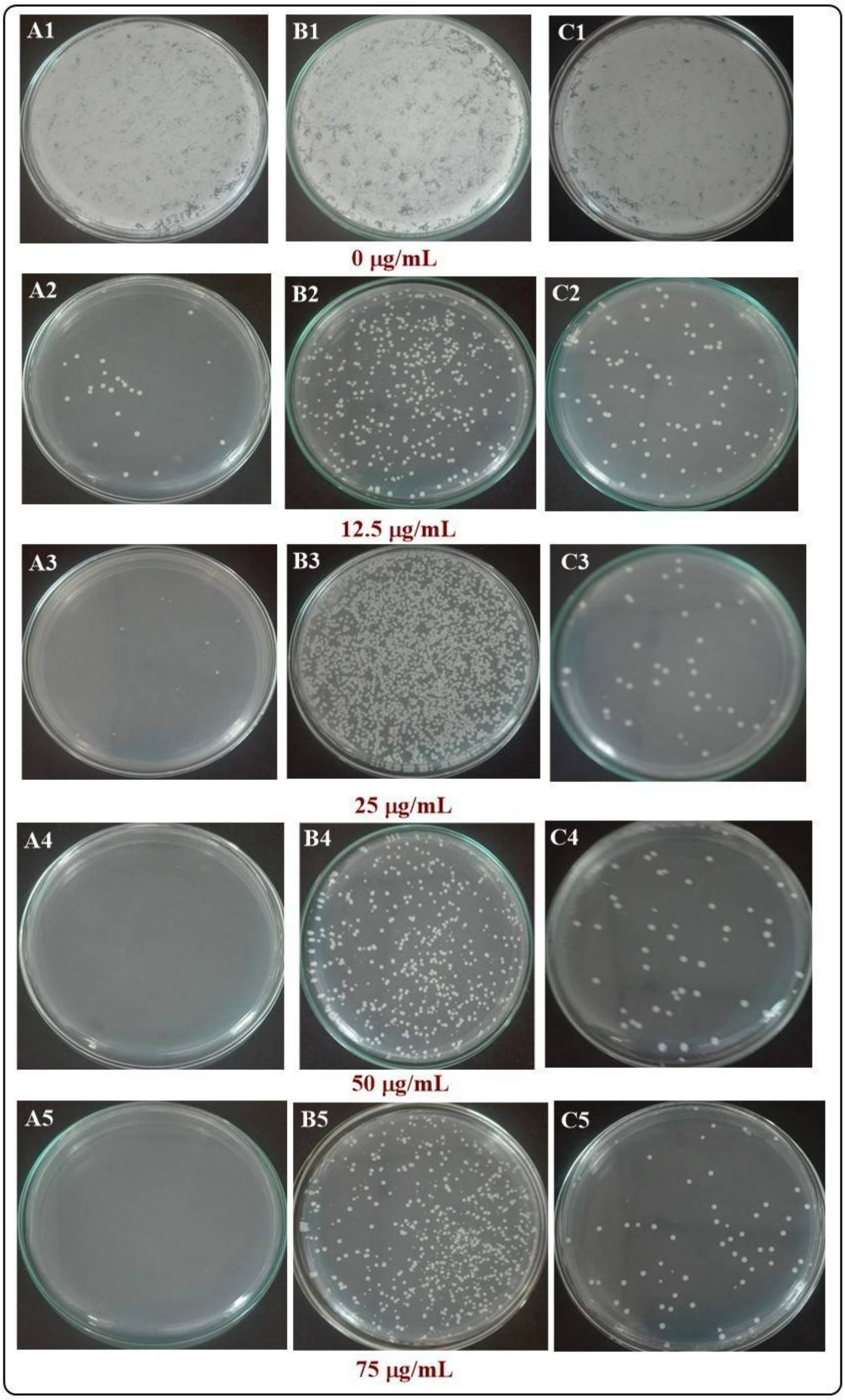

Figure S5. Evaluation of the antifungal activity (*C. albicans*) of Cu@AG (A), growth from dilution (10^{-3}) (B) at different doses.

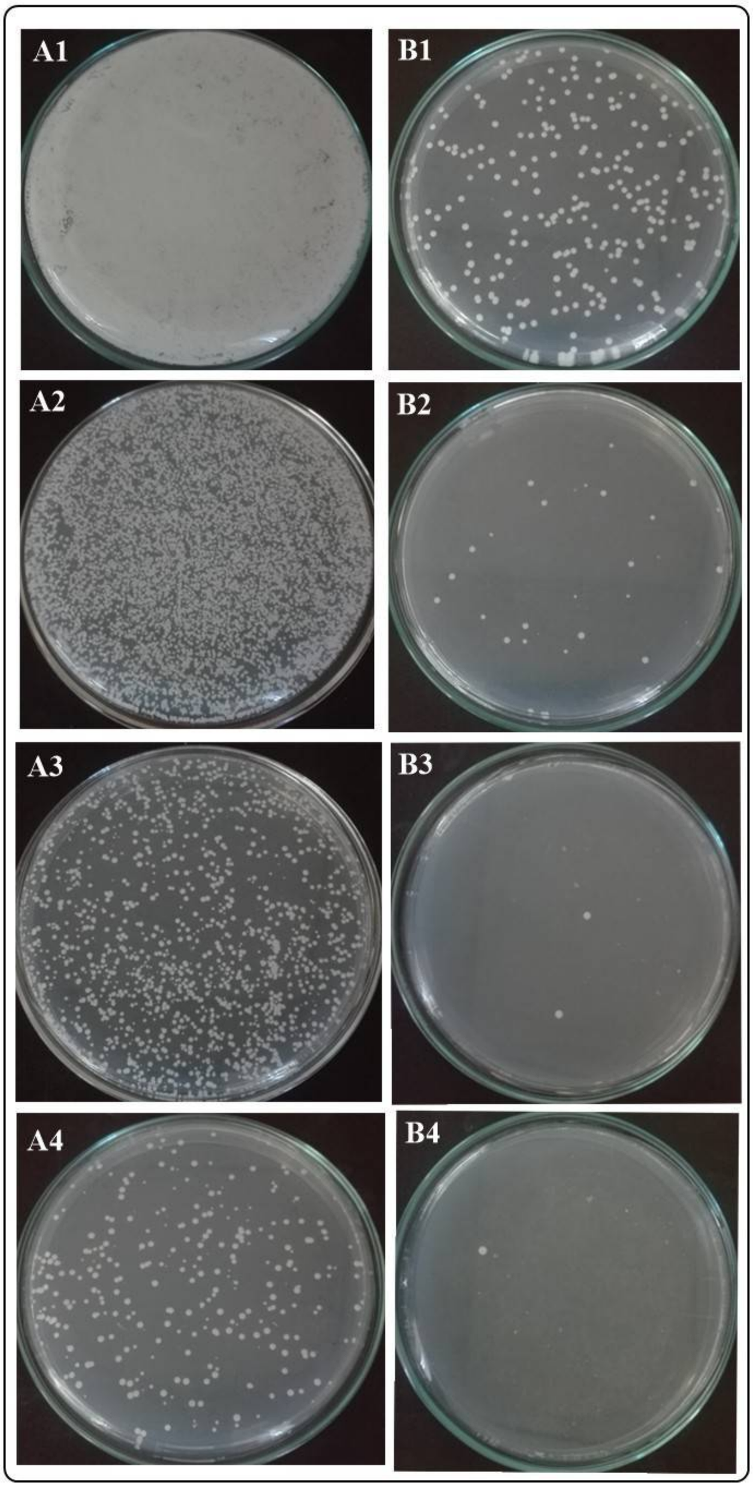

Figure S6. Evaluation of the antifungal activity of Cu@AG to inhibit *C.albicans* growth. A) Viability of control group (decimal dilutions) under experimental conditions (Top row). Evaluation of the fungicidal activity of Cu@AG against *C. albicans* (drop dilution test) at differen NPs concentrations. B) Log reduction on the CFU/mL of *C. albicans* exposed to different amounts of Cu@AG.

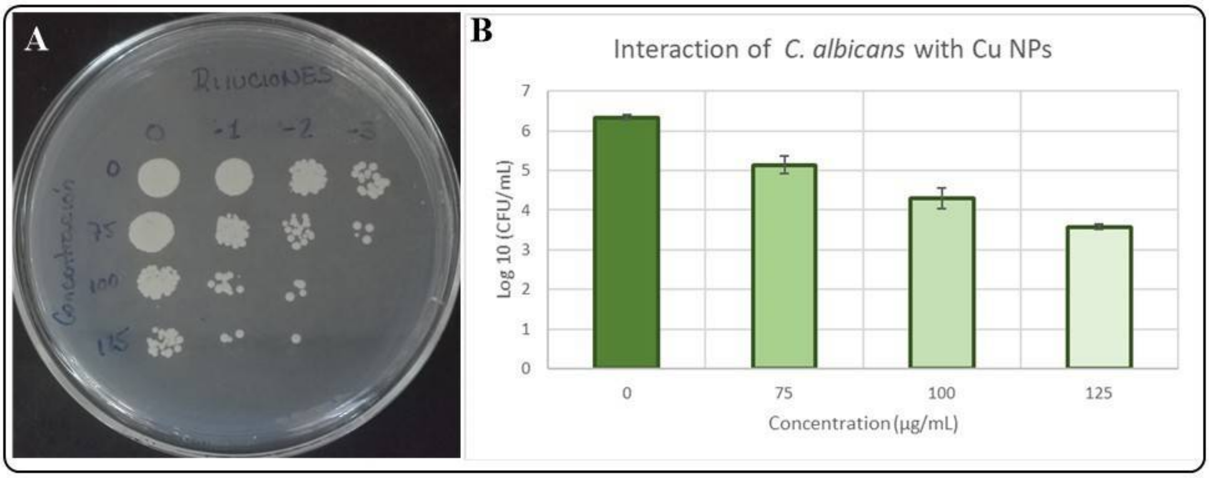

Figure S7. Evaluation of the antifungal activity (*S. schenckii*) of CuI@AG at different concentrations and exposition of 5h. A) Fungal cell are viable in the growth controls at different serial decimal dilutions under experimental conditions. B) Evaluation of the antifungal activity of CuI@AG by the drop dilution method. Fungal growth is only observed for growth control. C–F) Evaluation of the antifungal activity of CuI@AG by the pour plate method. Fungal growth is only observed for growth control (C).

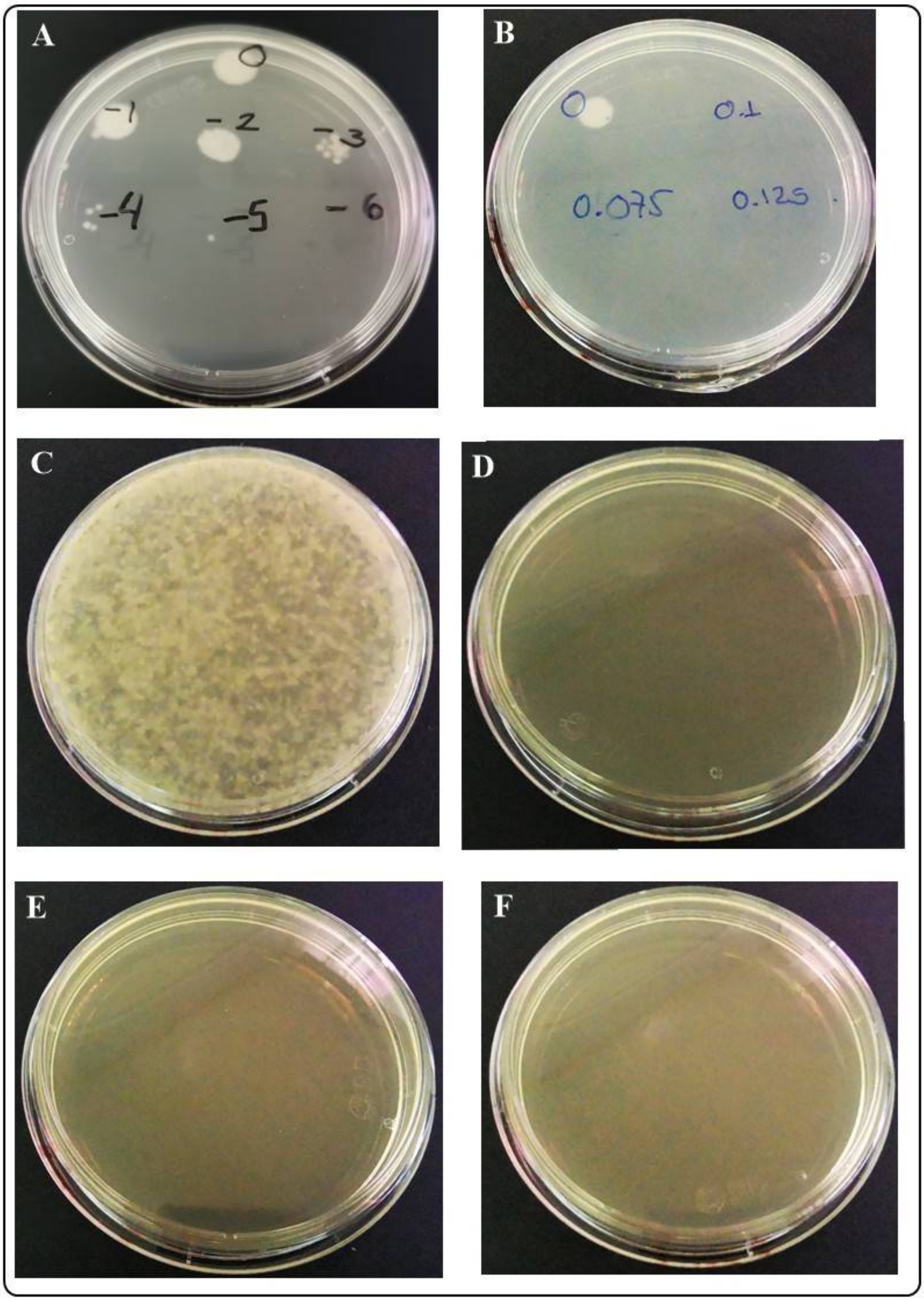

Figure S8. Evaluation of the antifungal activity (*S. schenckii*) of CuI@Ch at different concentrations and exposition of 5h. A) Fungal cell are viable in the growth controls at different serial decimal dilutions under experimental conditions. B) Evaluation of the antifungal activity of CuI@Ch by the drop dilution method. Fungal growth is only observed for growth control. C–F) Evaluation of the antifungal activity of CuI@Ch by the pour plate method. Fungal growth is only observed for growth control (C).

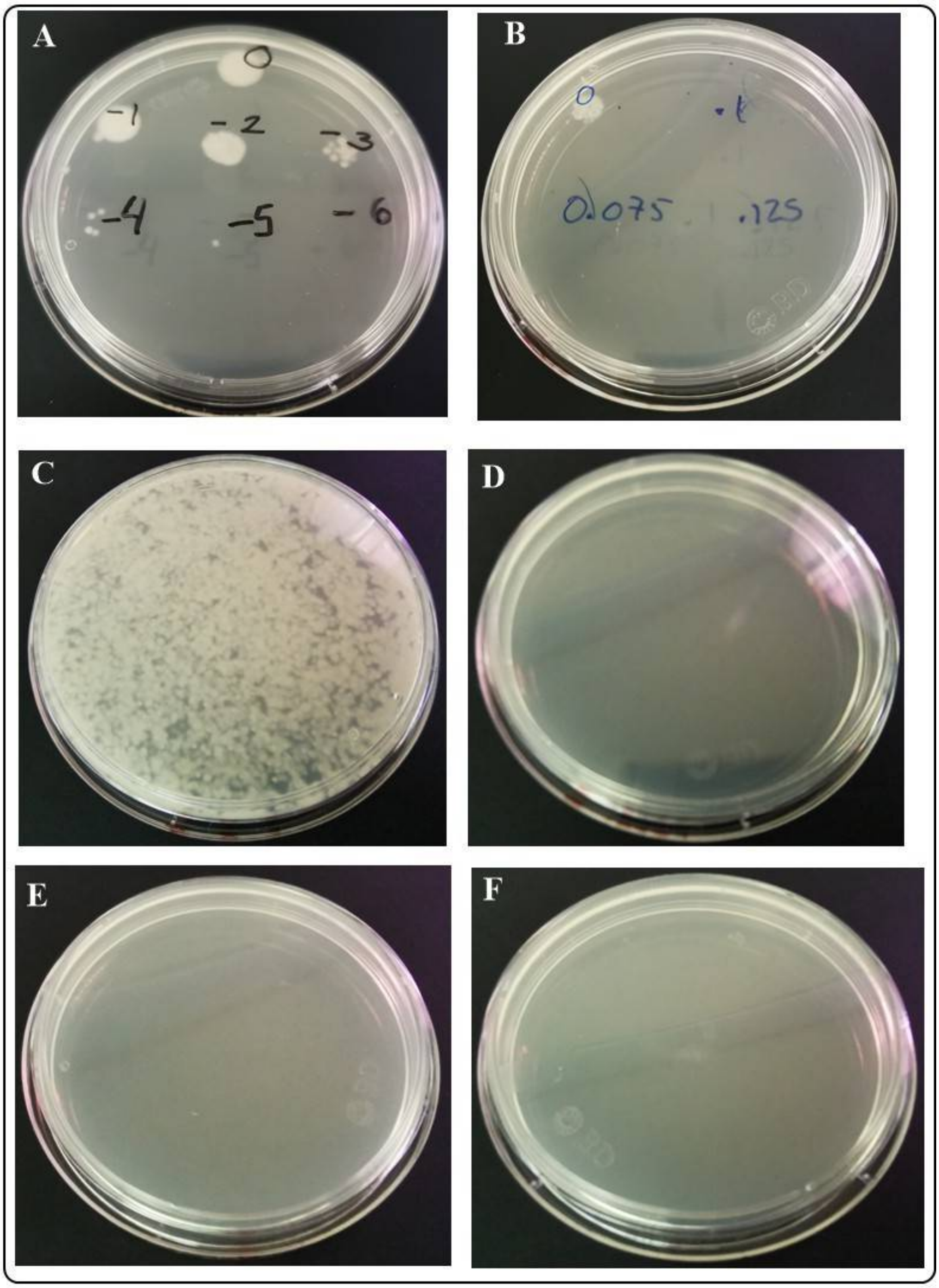

Figure S9. Evaluation of the antifungal activity (*F. oxysporumi*) of CuI@AG at different concentrations and exposition of 20h. A) Fungal cell are viable in the growth controls at different serial decimal dilutions under experimental conditions. B–D) Evaluation of the antifungal activity of CuI@AG at different doses by the drop dilution method. Fungal growth is only observed for the fungi exposed to 75 μg/mL of NMs.

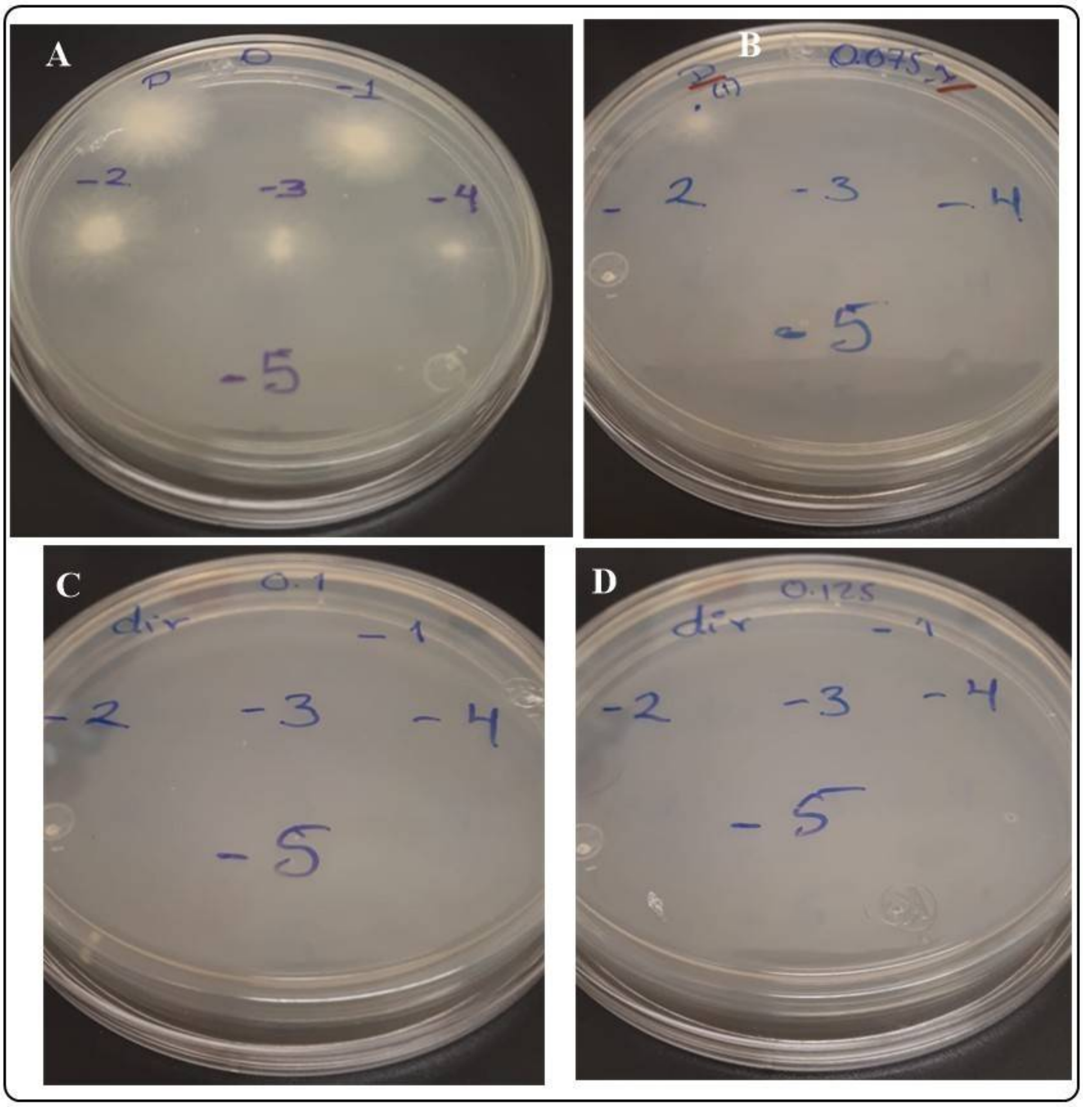

Figure S10. Optical microscopy examination of CuI-*C. albicans* interaction at diferent CuI doses. A) Big aggregates of *C. albicans* are observed for control group. B–D) The aggregates decrease as the concentration of NMs increases (50, 75 and 100 μg/mL respectively).

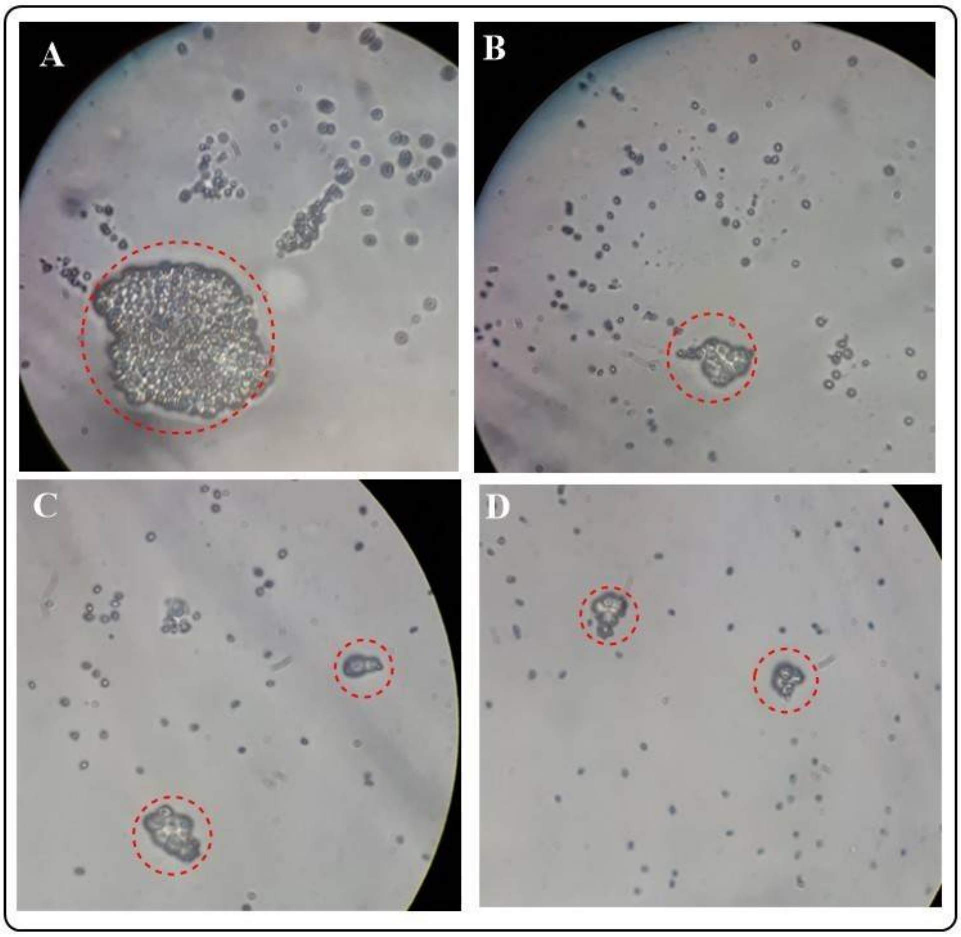

Figure S11. AFM analysis of the morphological changes in *C. albicans* due to exposure to CuI@AG (50 μg/mL x 1h). Formation of a pit in fungal cell is observed due to NMs penetration (red dotted circles).A–C are the low-magnification images, whereas D–F are the high magnification images.

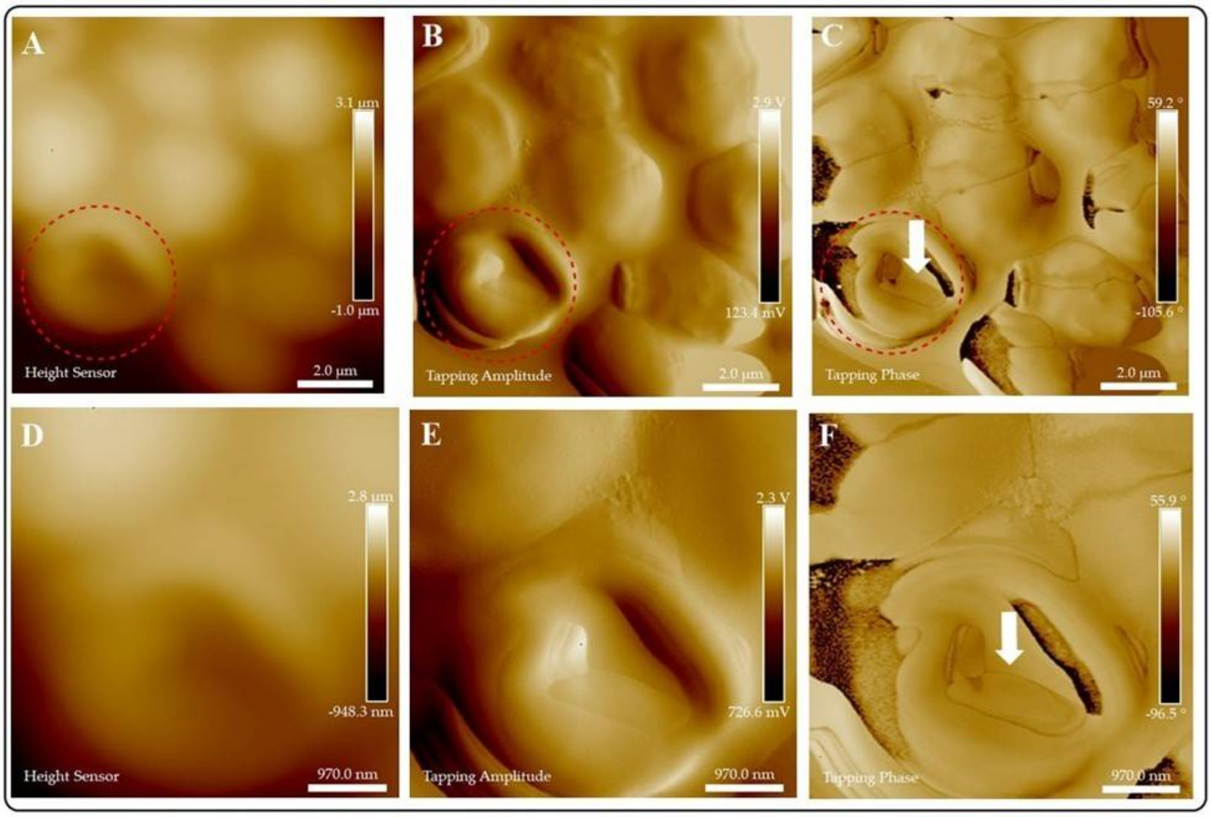

Figure S12. AFM analysis of the morphological changes in *S. schenckii* due to exposure to CuI@Ch (50 μg/mL x 5h). A–C) Control group. The fungi maintain their typical morphology under test conditions, demonstrating the bioavailability of the MOs at the end of the experiment. D-I) The NMs interact closely with fungal cells resulting in their destruction.

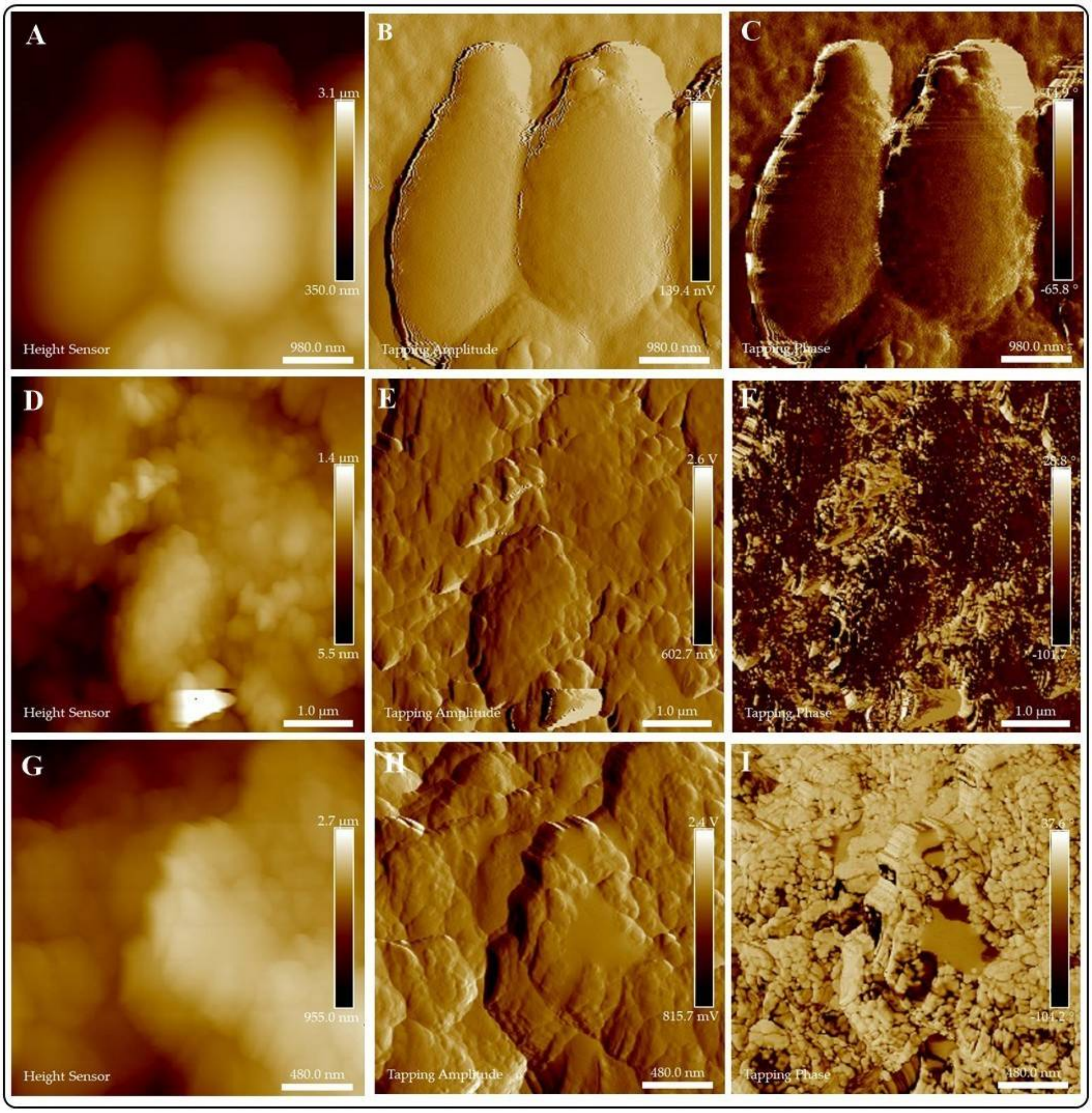

Supplement: Supplementary file 1 [file jof-07-00158-s001.pdf]
